# Mechanically adaptive materials with tunable stiffness for biomedical devices: Mechanisms, applications, and perspectives

**DOI:** 10.1016/j.mtbio.2026.103143

**Published:** 2026-04-19

**Authors:** Jiawen Xu, Run Lin, Dongfei Huo, Junyan Li, Oana R. Ghita, Ken E. Evans, Xijin Hua

**Affiliations:** a Department of Engineering, Faculty of Environment, Science and Economy, University of Exeter, Exeter, EX44QF, UK; b Department of Orthopedics, Shanghai University of Medicine & Health Sciences Affiliated Zhoupu Hospital, Shanghai, 201318, China; c School of Medical Instrument, Shanghai University of Medicine & Health Sciences, Shanghai, 201318, China

**Keywords:** Adaptive stiffness, Stimuli-responsive materials, Mechanotransduction, Compliance matching, Biomechanical modulation

## Abstract

Variable-stiffness materials are engineered systems that can reversibly tune their mechanical stiffness through diverse mechanisms. In recent years, researchers have paid growing attention to these materials because they can adapt to different tasks, support multiple functions, and safely interact with soft biological tissues. For these reasons, they show strong potential for biomedical devices. However, most existing studies focus on single material systems or individual actuation modes, which limits the integration of different stiffness-control strategies within metamaterial-inspired architectures. As a result, cross-mechanism synergy remains underexplored, and unified system-level design rules are still lacking. This review summarizes recent progress in variable-stiffness materials for biomedical devices over the past decade. We divide current approaches into three main groups based on their working mechanisms: jamming-based systems, stimuli-responsive material-based systems, and antagonistic actuation-based systems. For each group, we explain the basic working principles and typical structural designs. We then describe how different activation methods control stiffness and support functional adaptation. Building on this, an application-oriented analysis is conducted across five representative biomedical device domains, including drug delivery systems, wearable assistive devices, minimally invasive surgical tools, bone repair applications, and adaptive sensing platforms, with a comparative evaluation of the suitability and prevalence of different stiffness modulation mechanisms in each domain. Finally, we discuss future research directions. These include AI-based real-time stiffness control, multimodal human-machine interfaces, 4D-printed adaptive structures, and bio-integrated hybrid materials. Together, these advances may help move Variable-stiffness materials from laboratory studies toward practical clinical use.

## Graphical abstract

**Figure:**
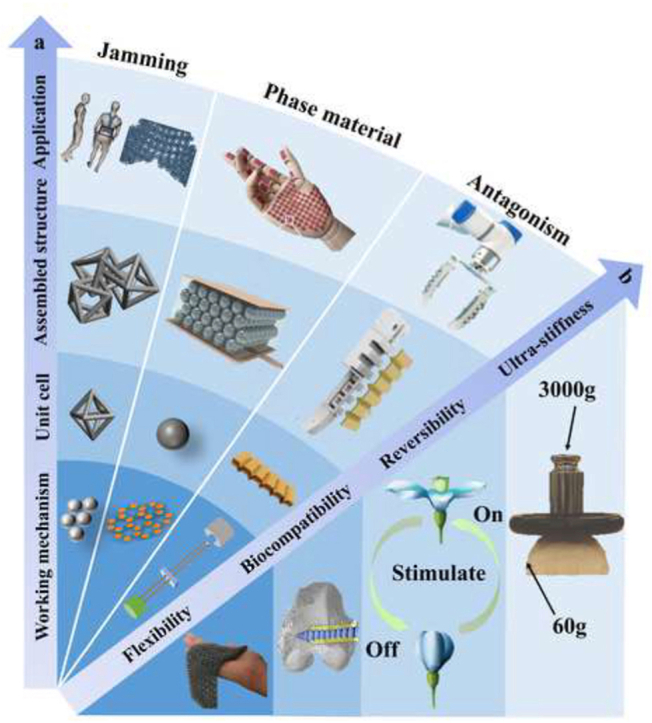

## Acronyms

VSMs: Variable-stiffness materials
MIS: Minimally invasive surgery
MJF: Multi Jet Fusion
SMs: Smart materials
LMPAs: Low-melting-point alloys
SMAs: Shape memory alloys
SMPs: Shape memory polymers
TPs: Thermoplastics polymers
ER: Electrorheological
MR: Magnetorheological
VST: Variable stiffness thread
MPTM: Magnetoactive phase-transitional matter
HIFU: High-intensity focused ultrasound
LMs: Liquid metals
MRI: Magnetic resonance imaging
HMI: Human-machine integration
PINNs: Physics-informed neural networks

## 1 Introduction

With the rapid development of biomedical technology, wearable devices, and minimally invasive tools, researchers increasingly need soft actuators and structural materials that can balance flexibility and load-bearing ability [[1], [2], [3], [4], [5], [6], [7]]. Conventional rigid medical devices often exert excessive or uneven forces when interacting with soft tissues, increasing the risk of tissue damage and limiting their adaptability to complex and dynamic environments [[8], [9], [10]]. This limitation makes it difficult to achieve accurate force transmission and precise positioning during procedures such as needle insertion, biopsy, and tissue manipulation [[11], [12], [13]]. As a result, biomedical devices face a long-standing trade-off between softness and stiffness. Devices must remain compliant during navigation but become stiff during operation. Similar stiffness-regulation strategies are widely observed in biological systems. For example, the dermis of sea cucumbers can rapidly and reversibly switch between soft and stiff states through changes in collagen crosslinking, enabling adaptive protection and locomotion [14]. Human skin also exhibits nonlinear stress-strain behavior, allowing it to remain compliant under small deformations while becoming significantly stiffer under larger loads. In the musculoskeletal system, antagonistic muscle-tendon structures regulate joint stiffness through coordinated contraction and relaxation [15,16]. Beyond these examples, many soft biological tissues also exhibit complex dynamic mechanical behaviors, including nonlinear elasticity, strain stiffening, viscoelastic relaxation, creep, and rate-dependent responses, which enable adaptive mechanical responses under varying physiological conditions [[17], [18], [19], [20]]. For instance, collagen-based tissues such as skin often display nonlinear stress-strain relationships and strain-stiffening effects, allowing them to remain compliant under small deformations while resisting larger loads [[21], [22], [23]]. In addition, biological tissues commonly exhibit viscoelastic properties, including stress relaxation and creep, which allow gradual redistribution of internal stresses over time. Their mechanical response is also rate-dependent, meaning that the effective stiffness can increase under rapid loading conditions. These dynamic mechanical characteristics provide important inspiration for the design of variable-stiffness materials capable of adapting to changing mechanical environments in biomedical applications [[24], [25], [26]]. Inspired by these biological mechanisms, stiffness modulation in synthetic materials can also arise from molecular and supramolecular processes, such as polymer chain mobility, glass-transition behavior, and reversible crosslinking networks [27,28]. These mechanisms are often governed by dynamic intermolecular interactions, including hydrogen bonding, ionic interactions, and dynamic covalent bonds, which enable reversible changes in network stiffness. In addition, architectures such as bottlebrush polymers and entropic elastic networks can further tune mechanical responses through chain conformation and entropy-driven elasticity, providing complementary pathways for achieving tunable stiffness in soft and biointegrated systems. These mechanisms provide additional inspiration for the design of adaptive materials capable of dynamic mechanical regulation. Despite these advances, which enable stiffness modulation through bioinspired and material-level mechanisms, these approaches are largely limited to intrinsic material responses or localized structural regulation, lacking cross-scale system integration. As a result, they cannot simultaneously meet the competing demands of compliance, load-bearing capacity, and functional stability in complex biomedical environments, and the fundamental trade-off between flexibility and stiffness remains unresolved.

Building on these advances, variable-stiffness materials (VSMs) have emerged as an important class of mechanically adaptive systems for biomedical applications. These systems often adopt metamaterial-inspired architectures and integrate mechanisms such as variable-stiffness composites, phase-change materials, and jamming-based structures. As a result, they can reversibly switch between soft and stiff states under different physiological conditions or task requirements [[29], [30], [31]]. This ability is especially important for minimally invasive surgery (MIS). In MIS, flexible tools such as endoscopes and catheters must move through complex and fragile anatomical regions [32,33]. At the same time, these tools must provide enough stiffness during operation to allow accurate and effective tissue manipulation [34,35]. For this reason, integrating VSMs into minimally invasive instruments may improve surgical accuracy and reduce intraoperative risk. This integration may also contribute to better patient outcomes [11,36].

Owing to these advantages, VSMs have attracted increasing attention in a wide range of biomedical applications beyond minimally invasive surgery, including soft robotics, prosthetic devices, and tissue engineering [[37], [38], [39]]. In robot-assisted surgery, variable-stiffness actuators can improve flexibility and control [[40], [41], [42]]. This improvement allows safer and more stable interaction with biological tissues [43]. In prosthetic systems, materials that adapt their stiffness in real time can better match the changing mechanical behavior of native tissues. As a result, these systems can improve wearing comfort and functional performance [[44], [45], [46], [47]]. In tissue engineering and regenerative medicine, materials with adjustable stiffness provide mechanical cues that influence cell growth, differentiation, and tissue remodeling. These cues can improve the performance of engineered scaffolds [48,49]. However, despite these application-level advances, many challenges still limit the clinical translation of variable-stiffness materials [50].

Although several review articles have summarized soft robotics, smart materials, or specific actuation strategies for stiffness-tunable systems, most existing reviews primarily focus on individual material platforms, actuator technologies, or specific device implementations. Consequently, the relationship between stiffness-regulation mechanisms and biomedical functional requirements has not been systematically analyzed. In particular, a systematic framework that compares different stiffness-modulation strategies from a materials-science and mechanistic perspective remains lacking. This limitation makes it difficult to evaluate how different mechanisms perform under realistic biomedical constraints such as activation methods, device geometry, biocompatibility, and clinical workflow integration. Future studies need to address several practical issues. These include faster and more reversible stiffness changes, improved biocompatibility with stable long-term performance, and fabrication methods that allow scalable and cost-effective manufacturing. To address these challenges and the increasing design complexity, computational modeling and machine learning tools have been increasingly adopted to assist material development. By establishing structure–property relationships, these approaches enable efficient optimization for application-specific requirements [51,52].

Building on this gap, the present review focuses on three major stiffness-regulation strategies and biomedical application requirements remains lacking. Building on this gap, this review examines recent research on variable-stiffness materials for biomedical devices. We focus on three main stiffness regulation strategies: jamming-based mechanisms, phase-change-based mechanisms, and antagonistic structural systems. Rather than treating these strategies as isolated material solutions, this review aims to establish a materials-science-informed design framework that links stiffness-regulation mechanisms to application-specific constraints in biomedical systems. Unlike existing reviews that typically focus on individual material classes or specific device implementations, this work provides a cross-mechanism comparison and links stiffness-regulation strategies to biomedical constraints. To achieve this goal, we summarize recent progress, identify unresolved challenges, and discuss emerging research opportunities. We pay particular attention to how different mechanisms affect system adaptability, operational accuracy, and biocompatibility. We also analyze the factors that currently limit clinical translation. This review is organized as follows. Section 2 reviews the development history of variable-stiffness materials. Section 3 introduces the three classes of stiffness regulation strategies and explains their basic working principles. Section 4 reviews representative applications in common biomedical scenarios. Section 5 compares insights across application domains to extract shared design principles and discuss future research directions. Summarizing existing work, this review argues that effective VSMs design does not depend only on the achievable stiffness range. Instead, performance depends on how well the selected regulation mechanism matches specific application requirements, such as activation methods, device geometry, sterilization conditions, and clinical workflow.

## 2 Debut, recent developments, and current situation

**Fig. 1:**
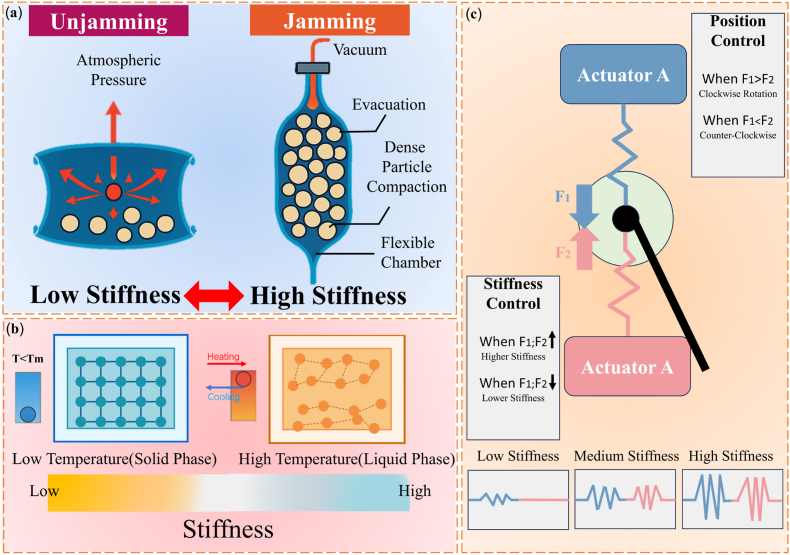
(a) The tunable stiffness mechanism of granular jamming. (b) The tunable stiffness mechanism of phase-change transition. (c) The tunable stiffness mechanism of antagonism.

VSMs originate from biomimetic ideas. Researchers first drew inspiration from biological systems such as muscles, tendons, and plant movements, where stiffness changes in response to functional needs [49]. Early engineering studies mainly focused on soft robotics and prosthetic devices. These studies aimed to reproduce the adaptive mechanical behavior of biological tissues by using stiffness-adjustable materials [53,54]. Based on these early explorations, several fundamental stiffness-regulation mechanisms have gradually emerged in VSM research. As illustrated in Fig. 1, jamming-based mechanisms regulate stiffness through particle confinement and frictional interactions; phase-change–based mechanisms rely on reversible solid–liquid transitions triggered by thermal stimuli; and antagonistic structural systems modulate stiffness through the force balance between opposing actuators. These representative mechanisms provide distinct physical pathways for achieving tunable stiffness in biomedical systems.

**Fig. 2:**
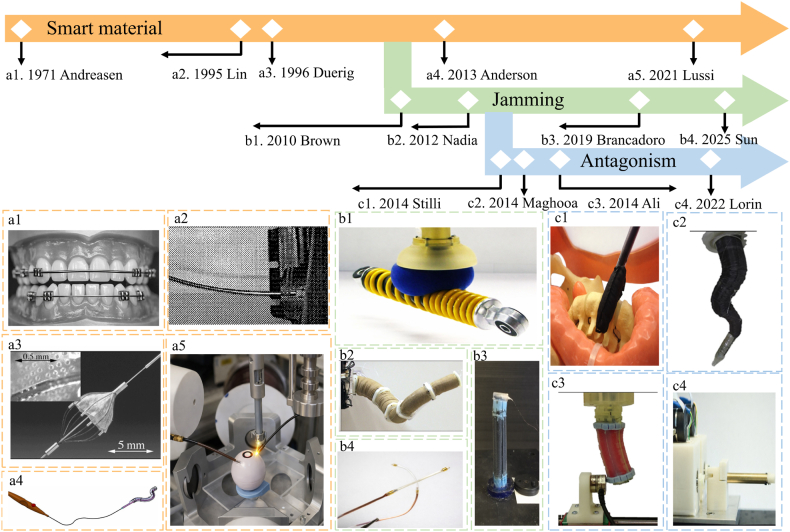
Timeline of representative variable-stiffness strategies in biomedical systems **(**a1) Nitinol archwire used in orthodontic alignment [55]. (a2) SMA-actuated bending of a surgical forceps [56]. (a3) Nitinol structure exhibiting superelastic deformation and recovery [57]. (a4) Schematic concept of the first generation Nickel Titanium Based Organ Positioner (NiTiBOP) [58]. (a5) Overview of the proposed catheter [59]. (b1) Granular jamming-enabled universal gripper showing stiffness transition for adaptive grasping [60]. (b2) Granular jamming-enabled continuum manipulator with tunable stiffness and high articulation [61]. (b3) Fiber jamming-enabled variable stiffness structure for surgical manipulation [62]. (b4) Fiber jamming-enabled variable stiffness catheter for minimally invasive intervention [63]. (c1) Antagonistic soft manipulator with tunable stiffness and shrinkable configuration [64]. (c2) Tendon-pneumatic antagonistic manipulator with tunable stiffness [65]. (c3) Tendon-stiffened pneumatic soft manipulator with tunable stiffness [66]. (c4) Antagonistic series elastic actuator for a robotic endoscope joint with tunable stiffness [67].

These mechanisms have undergone continuous development from early conceptual demonstrations to clinically relevant implementations, as summarized in Fig. 2. Smart material-based variable stiffness systems in biomedical devices have evolved from passive material responses to actively controllable and miniaturized implementations. Early applications relied on the superelastic behavior of NiTi alloys in orthodontics, enabling large reversible deformation and relatively stable force output (Andreasen, 1971) [55]. In the 1990s, this behavior was further linked to medical functionality, while shape memory alloy (SMA)-based actuation was introduced into minimally invasive surgical tools such as laparoscopic forceps (Nakamura et al., 1995; Duerig et al., 1996), marking a transition toward active stiffness control [56,57]. Subsequent developments enabled explicit stiffness tuning through phase transformations, and more recent advances have achieved continuous and miniaturized stiffness regulation at submillimeter scales, exemplified by variable-stiffness catheters (Anderson et al., 2013; Lussi et al., 2021) [58,59]. Overall, these systems demonstrate a clear progression from passive mechanical response to actively controlled and application-oriented stiffness regulation. In parallel, jamming-based variable stiffness systems have developed from fundamental physical demonstrations toward practical biomedical implementations. Early studies established granular jamming as a mechanism for stiffness modulation, where particulate media transition between fluid-like and solid-like states under vacuum (Brown et al., 2010) [60]. This principle was subsequently extended to articulated and continuum manipulators enabled by granular media, demonstrating its feasibility in flexible robotic structures (Cheng et al., 2012) [61]. Later work introduced fiber-based jamming strategies for surgical manipulators, providing improved controllability and structural adaptability in confined environments (Brancadoro et al., 2019) [62]. More recently, jamming mechanisms have been integrated into miniaturized biomedical devices, such as cardiovascular catheters, enabling rapid and reversible stiffness tuning for safe navigation and intervention (Sun et al., 2025). This evolution reflects a transition from physical principle validation to clinically relevant, miniaturized systems. Meanwhile, antagonistic variable-stiffness systems have progressed from bio-inspired actuation concepts toward integrated control and clinical applicability. Early work introduced antagonistic configurations combining pneumatic expansion and tendon contraction to achieve simultaneous shape morphing and stiffness modulation in soft manipulators for minimally invasive surgery (Stilli et al., 2014) [64]. This concept was subsequently refined through hybrid tendon–pressure actuation frameworks, enabling improved control over workspace and stiffness regulation (Maghooa et al., 2015) [65]. Further developments translated this principle into dedicated stiffening strategies for pneumatically actuated soft manipulators, highlighting its effectiveness in balancing compliance during insertion with rigidity during operation in constrained surgical environments (Shiva et al., 2016) [66]. More recent studies have extended antagonistic mechanisms to robotic endoscope joints using series elastic actuation, where stiffness modulation is coupled with force estimation and impedance control to enhance safety and interaction performance (Fasel et al., 2022) [67]. Collectively, these systems demonstrate a shift from conceptual bio-inspired designs to controllable, application-driven solutions for safe and precise medical manipulation.

**Fig. 3:**
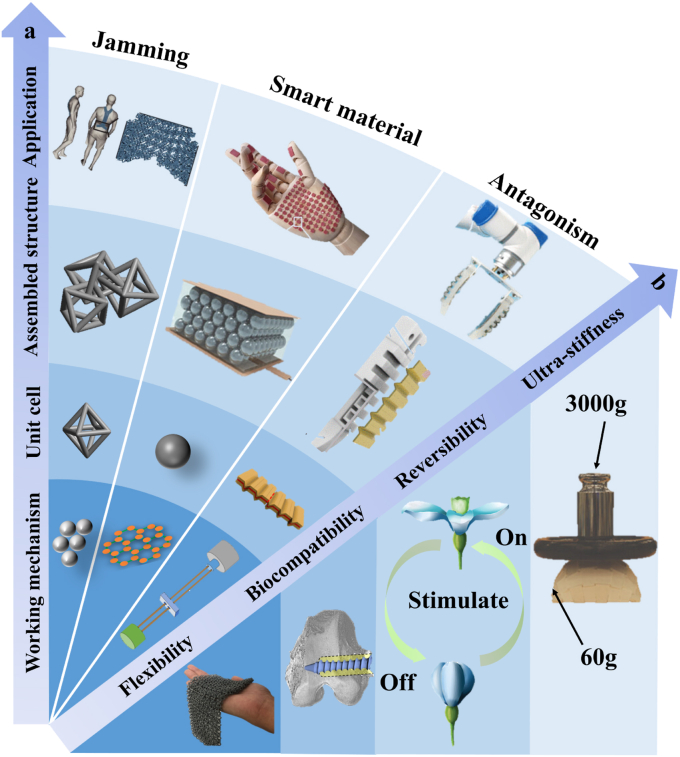
(a) Overview of stiffness-tuning approaches: unit cell, assembled structures, and their potential applications [[68], [69], [70]]. (b) The excellent performance of VSMs [6,[71], [72], [73]].

These developments can be systematically organized into a hierarchical design framework across multiple scales. As illustrated in Fig. 3a, variable-stiffness systems are constructed through a hierarchical, multi-scale design process, starting from fundamental working mechanisms, followed by the development of unit-cell architectures, which are subsequently assembled into integrated structures and further incorporated into functional biomedical devices with tunable stiffness. This structural hierarchy provides the basis for achieving diverse functional properties in biomedical applications, as summarized in Fig. 3b. At present, VSMs are increasingly used in biomedical systems because they combine flexibility, reversibility, biocompatibility, and adjustable mechanical stiffness [6,[71], [72], [73]]. These properties are important for biomedical use. Such materials must adapt to changing physiological conditions and reduce mechanical mismatch with biological tissues. At the same time, they must maintain stable and safe performance during long-term operation. When integrated into medical devices, prosthetic systems, and tissue engineering scaffolds, VSMs can improve patient comfort and mechanical performance. They can also support new therapeutic approaches. For these reasons, VSMs provide a practical material basis for the development of next-generation biomedical technologies.

Despite recent progress, VSMs still face major barriers when moving from laboratory prototypes to clinical systems. Current limitations include low energy efficiency, limited long-term durability, difficulties in biocompatible encapsulation, and the need for complex and bulky control hardware. In many reported systems, stiffness changes take several hundred milliseconds to a few seconds. These systems often require strong thermal, pneumatic, or magnetic inputs. Such requirements reduce portability and make continuous operation difficult. For these reasons, overcoming these challenges is essential before VSMs can move beyond proof-of-concept studies and be used in practical biomedical devices.

## 3 Strategies for tuning stiffness

### 3.1 Jamming-based structural design

Jamming-based variable stiffness systems control stiffness through geometry and contact interactions between structural elements. Unlike many smart material systems, jamming mechanisms do not rely on nonlinear material behavior. Instead, stiffness emerges from frictional interactions and geometric confinement between internal particles, layers, or fibers. This design enables a large stiffness change while maintaining a lightweight and porous structure [74]. Such features are particularly suitable for biomedical applications, where devices must remain soft and compliant during placement and navigation but become stiff during deployment or force transmission [75]. In jamming-based systems, stiffness modulation typically depends on external confinement. Applied pressure, vacuum, or mechanical locking increases the contact forces between internal elements, forming a jammed network that restricts relative motion. As a result, the structure transitions from a loose and mobile state to a constrained load-bearing configuration, allowing reversible switching between soft and stiff states [36].

In recent years, progress in architected mechanical metamaterials has expanded the design options for jamming-based VSMs. Many studies now use lattice-like or textile-like structures. These structures consist of connected struts, plates, or hollow units, with characteristic sizes ranging from sub-millimeter to centimeter scales. Researchers design these architectures using computational topology optimization and fabricate them with high-resolution additive manufacturing methods. By adjusting unit cell geometry, contact surface properties, and interlocking patterns, researchers can control when jamming occurs and how much stiffness change is achieved. These design choices also affect directional stiffness behavior. At the same time, the structures can remain lightweight and compact, which meets the size and weight limits required for biomedical applications. Because these jamming effects depend strongly on precise geometric features and reproducible contact conditions, fabrication methods must be able to accurately realize thin walls, hollow units, and interlocking interfaces.

**Fig. 4:**
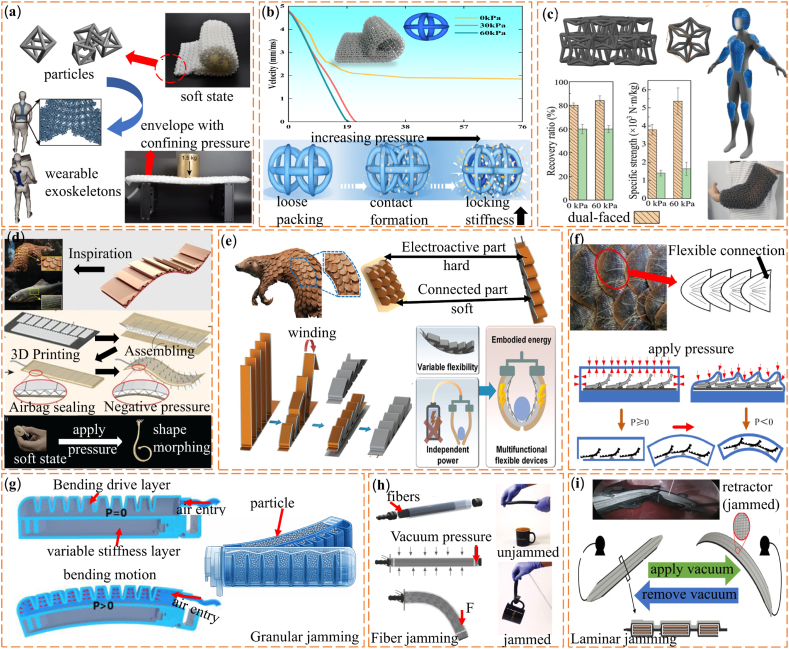
(a) Architected chain-mail-like textile composed of interlocking hollow units enabling pressure-induced jamming [68]. (b) Dynamic response of chain-mail fabrics under different confining pressures [73]. (c) Dual-faced architected textile with tunable mechanical performance [77]. (d) Bioinspired scale-like layered structure (SAILS) with geometry-controlled locking [78]. (e)Overlapping scale-inspired structure with tunable stiffness [79]. (f) Pangolin-inspired variable stiffness mechanism [80]. (g) Hybrid particle-based jamming structure for soft grasping [81]. (h) Fiber jamming mechanism in slender structures [82]. (i) Laminar jamming-based soft retractor for minimally invasive applications [83].

To realize these architected textile geometries in practice, suitable additive manufacturing methods are required. Among them, Multi Jet Fusion (MJF) printing with PA12 provides a practical route for fabricating architected textile structures, because it enables relatively precise control of wall thickness, porosity, and interlocking geometries while maintaining consistent mechanical performance across samples [76]. Wang et al. [68]. reported a chain-mail-like textile made of two interlocking layers of hollow units. In this structure, each unit acts as an effective particle within the overall system. The interlocking geometry increases the number of potential contact interfaces, so that external pressure rapidly converts discrete contacts into a dense load-bearing network. When an external pressure of 93 kPa was applied, contacts and jamming between neighboring units increased the bending stiffness by more than 25 times. This change of stiffness reflects a pressure-driven transition. The measured mechanical response follows a power-law relation similar to that observed in assemblies of convex granular materials. Because of this behavior, such architected textile systems are suitable for shape-adaptive exoskeletons, reconfigurable support structures, and adaptive tactile interfaces, as shown in Fig. 4a.

Building on chain-mail concepts, Xu et al. [73]. developed a pressure-sensitive architected textile system using geometrically customized particle units. In this design, the particle geometry increases the number of potential contact points and enhances interlocking between neighboring units under confinement. As a result, the contact network becomes denser during compression, which amplifies frictional resistance and leads to a significant increase in stiffness. When a confining pressure of 60 kPa was applied, the specific strength increased … from 2.20 to 9.19 N m kg^−1^, which corresponds to a 4.2-fold increase. The peak impact load rose by more than four times. At the same time, the apparent bending modulus and energy absorption capacity increased by over 20 times and 22 times, respectively, as shown in Fig. 4b. To address the trade-off between high specific strength and structural recoverability, Tian et al. [77]. proposed a double-sided chain-mail-like structure based on three-dimensional re-entrant units and a new interlocking mechanism. This system achieved a specific energy absorption of 1530 J kg^−1^ and a specific strength of 5900 N m kg^−1^. It also showed a deformation recovery ratio of about 80%. These results indicate clear advantages for wearable protective systems that require both shape adaptability and high protective performance, as shown in Fig. 4c. Across these studies, moderate confining pressure is sufficient to trigger a reversible transition from a soft and mobile state to a rigid load-bearing state. This response provides a practical way to address the long-standing soft-rigid trade-off in wearable and rehabilitative devices [84].

In addition to chain-mail-like textiles, many bioinspired architected systems also use jamming-related transitions to achieve tunable stiffness. These designs typically rely on geometry-controlled contact locking, where overlapping or articulated elements progressively engage under external confinement. One common example is layered structures inspired by fish scales. These systems consist of overlapping rigid plates embedded in a soft matrix [78]. When negative confining pressure is applied, the plates lock into fixed positions. As a result, the bending modulus can change by up to 53 times at operating frequencies of up to 5 Hz. This behavior supports rapid reconfiguration and is useful for biomedical support structures, as shown in Fig. 4d. Scale-inspired designs have also been integrated into flexible lithium-ion batteries. In these systems, the structure provides energy storage, load-bearing ability, and stiffness tuning at the same time. This integration enables conformable power modules for future wearable medical devices, as shown in Fig. 4e [79]. In a related approach, pangolin-inspired soft grippers combine scale-like units with pneumatic actuation. These grippers show more than a twofold increase in stiffness compared with standard pneumatic grippers, while still adapting to objects of different shapes, as shown in Fig. 4f [80]. Other bioinspired designs further support this concept. Dragon-skin-inspired structures use vacuum-induced locking to fix shape when needed [85]. Snake-like pneumatic robots developed for natural orifice transluminal endoscopic surgery also rely on geometric reconfiguration and contact engagement to adjust stiffness during operation [86]. Together, these studies show that effective in situ stiffness control can be achieved by coordinating structural geometry and contact interactions.

At the material scale, jamming transitions have been widely studied in granular, fibrous, and layered systems. Many studies treat jamming as a robust and biocompatible way to achieve variable stiffness without adding complex external components. Since the first jamming-based gripper was reported in 1978 [87], this approach has developed steadily within biomedical soft robotics. In granular jamming systems, particles are enclosed in an elastic membrane. When vacuum or mechanical compression is applied, the particles change from a loose and mobile state to a solid-like state. This transition occurs because increasing confinement strengthens frictional contacts and creates a percolating force chain network throughout the particle assembly. Together, these effects form a stable contact network that resists deformation [88]. Researchers have applied this principle to hybrid designs that combine folding plate structures with local granular jamming. These designs create finger-like grippers with adjustable stiffness and reliable load-bearing ability under different loading directions, as shown in Fig. 4g [81]. Granular jamming systems are now used in several biomedical applications. Examples include robotic medical grippers [[89], [90], [91], [92], [93]], impact absorption pads, and reconfigurable support structures [60,94,95].

Granular jamming structures mainly provide high load-bearing capacity under compression and shear. However, many biomedical devices, especially slender instruments, also need to carry tensile loads along the axial direction. Fiber jamming was introduced to meet this need. Compared with granular particles, elongated fibers generate larger contact areas and directional frictional constraints when confined. In fiber jamming systems, elongated filaments are packed within a flexible sleeve so that confinement increases frictional contact along the fiber length. In the jammed state, the structure can resist tensile loads. When the constraint is released, the system remains flexible and easy to bend. Studies on cylindrical fiber jamming devices show that these structures offer a wide and controllable range of bending stiffness. They are suitable for uses such as surgical manipulators and wearable haptic feedback devices [62,96]. Laminar jamming follows a similar idea but uses stacked flexible sheets instead of fibers. When vacuum is applied, friction between adjacent layers increases dramatically, effectively transforming the stack into a unified bending-resistant laminate. As a result, bending stiffness often increases by more than one order of magnitude. In the unjammed state, the structure keeps good foldability, which supports minimally invasive insertion. Laminar jamming designs have been used in foldable soft retractors for laparoscopic surgery and natural orifice transluminal endoscopic surgery. In these procedures, instruments must meet strict limits on size and shape during insertion, deployment, and removal [97].

Jadhav et al. [82]. developed a slender fiber jamming module that allows continuous adjustment of bending stiffness across a wide range, as shown in Fig. 4h. This module is suitable for soft robotics and wearable haptic feedback systems, where both flexibility and controllable stiffness are required. The design demonstrates how fiber jamming can support tensile load bearing while maintaining good bending compliance. Beyond fiber jamming, laminar jamming has also been used in hollow and slender instruments for minimally invasive surgery. These mechanisms support controlled deployment and stiffness increase after insertion. Researchers have further applied laminar jamming to deployable structures and reconfigurable furniture systems. These examples show that laminar jamming performs well in confined spaces and in architectures that require shape reconfiguration [98].

Fig. 4i shows an engineered foldable laminar jamming soft retractor designed for conventional laparoscopic surgery. The study examines key design constraints related to instrument insertion, deployment, and retrieval. It shows that these requirements restrict each other and together define the feasible design space of laparoscopic variable-stiffness retractors [83]. Laminar jamming structures can typically change stiffness by more than one order of magnitude. Reported stiffness values range from about 0.01 to 1.5 N mm^−1^, while maintaining sufficient bending resistance. In recent years, researchers have integrated laminar jamming structures into haptic feedback devices [99,100] and medical instruments [101,102]. Their reversible stiffening behavior, slender form, and compatibility with minimally invasive deployment make them suitable for surgical robotic systems and human-machine interaction platforms.

In summary, jamming-based systems offer reversible, high-contrast stiffness switching through geometry-driven contact interactions, enabling lightweight, compact designs well-suited for biomedical applications. Their primary advantages include rapid pressure-controlled actuation, material-independent stiffness modulation, and versatile implementation across granular, fibrous, and laminar architectures. However, key limitations remain performance relies on precise fabrication and contact durability; absolute stiffness and load capacity are constrained by structural porosity; and most configurations require external confinement sources, adding system complexity. Future advances depend on improved manufacturing robustness, higher load capacity, and integrated actuation to expand clinical applicability.

### 3.2 Smart materials: phase-change and rheological systems

Smart materials (SMs) are materials whose mechanical properties can change in response to external stimuli such as temperature, light, electric fields, magnetic fields, or surrounding solvents [103,104]. In biomedical applications, these materials enable stiffness control at the material level. This feature supports variable-stiffness functions in systems such as drug delivery devices, minimally invasive surgical tools, actuators, and implantable components [105,106]. In biomedical variable-stiffness systems, smart materials can be broadly discussed in three subcategories: phase-change materials, field-responsive rheological materials, and other stimulus-responsive systems.

#### 3.2.1 Phase-change materials

Phase-change materials exhibit pronounced stiffness variation when undergoing solid–liquid transitions or when thermal stimuli alter polymer chain mobility. In these systems, stiffness modulation arises from changes in the internal molecular structure of the material, which significantly affect the elastic modulus and mechanical response. Common examples include low-melting-point alloys, shape memory alloys, shape memory polymers, and thermoplastic polymers. Although these materials share the common feature of stiffness modulation through temperature-driven phase transitions or molecular mobility changes, they differ significantly in stiffness contrast, activation temperature, response speed, and biomedical compatibility. These materials switch between compliant and stiff states through Joule heating, photothermal heating, or changes in the surrounding environment. Because stiffness control occurs within the material itself rather than through external structural constraints, phase-change materials are well suited for slender medical devices. Examples include catheters and steerable puncture needles, where space is limited and external mechanical components are difficult to integrate.

#### 3.2.2 Low-melting-point alloys

**Fig. 5:**
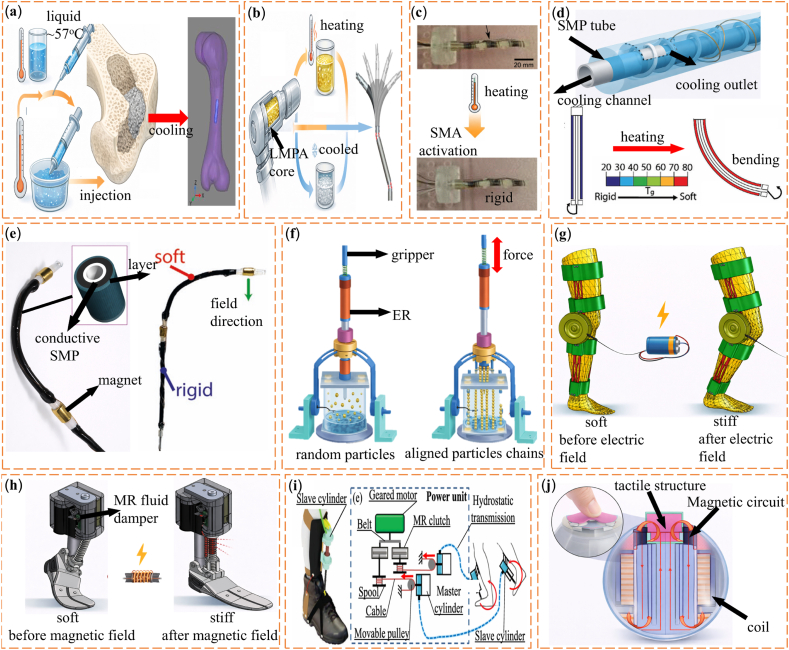
(a) Radiation imaging of porcine femur with alloy cement [115]. (b) Concept of a laparoendoscopic single-site surgery (LESS) manipulator with thermally tunable stiffness [116]. (c) Spring-based continuum robot demonstrating stiffness modulation via SMA actuation before and during heating [117]. (d) Fast-response variable-stiffness thread (FRVST) illustrating thermal activation and rapid stiffness transition [118]. (e) Conductive shape memory polymer (CSMP)-based variable stiffness thread (VST) showing reversible soft–rigid switching under external stimulus [119]. (f) Schematic of the proposed haptic master combined with the ER spherical haptic device and ER linear haptic device [120]. (g) An active knee rehabilitation orthotic device equipped with an ER brake [121]. (h) Assembly of the prototype of ankle and foot prosthesis [122]. (i) Wearable ankle exoskeleton with dual MR clutches enabling adaptive torque transmission and load redistribution through stiffness control [123]. (j) Spherical MR haptic sensor with an optimized magnetic circuit, capable of reproducing soft-tissue-like deformation via field-dependent rheological response [124].

Low-melting-point alloys (LMPAs) with melting temperatures below 300 °C show large and reversible stiffness changes while maintaining metal-level strength. Common systems include gallium-based and bismuth-based alloys. Gallium-based alloys form a thin oxide layer in air. This layer improves interfacial adhesion and mechanical stability. As a result, stiffness can be adjusted by controlling interfacial contact and microstructural arrangement [107]. Bismuth-based alloys offer clear phase transitions together with good thermal and electrical conductivity. These properties make them suitable for shape-adaptive implants and reconfigurable structural components [108,109]. Their high conductivity also allows rapid phase switching under relatively low energy input. This feature supports precise and repeatable stiffness control in applications that require fast response [59,[110], [111], [112], [113], [114]]. One representative example is an injectable Bi-In-Sn-Zn alloy bone cement with a melting point of about 57.5 °C. This material has been proposed as an alternative to polymethyl methacrylate bone cement for filling bone defects [115]. Near physiological temperature, the alloy changes from liquid to solid. This transition allows in situ shaping to match the defect geometry, followed by solidification into a load-bearing metallic scaffold. The material also provides good radiopacity, as shown in Fig. 5a. Beyond bone repair, LMPA cores have been used in flexible joints for exoskeletons and robotic arms. By selectively melting and solidifying the alloy inside the joint, stiffness can be tuned while preserving load-bearing capacity and structural reconfiguration [125,126]. Building on this concept, Wang et al. [116]. developed a robotic system in which each joint contains an LMPA core. Thermal switching between solid and liquid states enables on-demand control of the overall arm stiffness, as shown in Fig. 5b. Overall, these studies show that stiffness control using LMPAs depends strongly on structural geometry, heating strategy, and thermal management. These factors must be designed together to meet the needs of specific biomedical applications [127]. However, the relatively high melting temperatures of many LMPAs and the presence of metallic elements may raise concerns regarding thermal safety and long-term biocompatibility in direct-contact biomedical environments.

#### 3.2.3 Shape-memory materials

Shape memory alloys (SMAs) and shape memory polymers (SMPs) can store a permanent shape and be set into a temporary form. They return to the original shape when exposed to heat, electricity, or other external stimuli [128]. In SMPs, stiffness control usually occurs when the material passes its glass transition temperature. This change is often triggered by embedded heaters or photothermal additives. In contrast, SMAs rely on a martensite to austenite phase transformation. This transformation changes both shape and stiffness at the same time. Because of their good biocompatibility and mechanical durability, SMAs have been used in biomedical devices for many years [129]. Early engineering use of shape memory materials dates back to a 1941 patent on elastic memory resin for dental applications, which established the basis for later biomedical adoption [130]. In neurosurgery, Kim et al. [117]. developed a flexible continuum robot that uses an SMA-based stiffening mechanism. The robot remains compliant during navigation and becomes rigid during operation, which improves positioning accuracy, as shown in Fig. 5c. SMP-based variable stiffness mechanisms have also been applied to minimally invasive surgical tools. For example, researchers integrated a fast-response SMP thread into catheter systems. This design allows the catheter to stay flexible during navigation and stiffen quickly when needed, as shown in Fig. 5d [118]. Later studies introduced electrically conductive SMP composites. In these materials, Joule heating raises temperature directly inside the polymer. This approach removes the need for external heaters and allows segmented stiffness control in magnetically assisted catheters, as shown in Fig. 5e [119]. Despite these advantages, thermal activation remains a major limitation in biomedical use. Compared with metallic SMAs, SMPs generally offer better flexibility and easier integration into soft biomedical devices, but they typically provide lower stiffness contrast and slower thermal response. SMPs and SMAs often require temperatures above their activation points. These temperatures can approach or exceed about 41 °C, which is close to the safety limit for biological tissue. In addition, cooling after activation can be slow and may reduce real-time control performance. For these reasons, progress toward clinical use depends on improved thermal management and the development of SMP and SMA systems that activate at lower temperatures.

#### 3.2.4 Thermoplastic polymers

Thermoplastic polymers (TPs) soften above their glass transition temperature ($T_{g}$) and revert to their original state upon cooling without chemical degradation [131,132]. Their wide modulus range across low cost, and compatibility with 3D printing make them attractive for variable-stiffness structures. Thermoplastic systems are increasingly explored for biomedical variable-stiffness structures because they combine low cost, processability, and compatibility with additive manufacturing. Examples include thermoplastic starch-based cellular structures inspired by deep-sea glass sponges for MIS tools [133], polycaprolactone (PCL)-based grids embedding distributed sensing and actuation for locally tunable stiffness [134], and thermoplastic polyurethane (TPU)-SMA hybrid grippers that combine compliant TPU backbones with thermally activated SMA wires for stiffness modulation [135]. The main limitation of thermoplastic-based stiffness tuning is slow thermal response, typically taking 30-90 s for millimeter-scale sections which is considerably slower than the sub-second response desired in many surgical and haptic applications.

Overall, different phase-change materials offer distinct advantages for biomedical variable stiffness systems. LMPAs provide large stiffness contrast and metallic load-bearing capability but may raise concerns related to thermal safety and metal ion exposure. SMAs offer high actuation force and compact integration but often suffer from thermal lag and fatigue under repeated phase transitions. SMPs and thermoplastic polymers generally exhibit better flexibility and easier integration into soft biomedical devices, although their achievable stiffness change and response speed are typically lower. Therefore, selecting an appropriate phase-change material requires balancing stiffness range, activation temperature, response time, and long-term biocompatibility for specific biomedical applications.

#### 3.2.5 Field-responsive rheological materials

Field-responsive rheological materials include electrorheological fluids and magnetorheological fluids. These materials change their viscosity and yield stress when an electric or magnetic field is applied. Under an external field, suspended particles rapidly align and form chain-like structures along the field direction, which restrict fluid motion and increase the effective stiffness of the system. As a result, these materials can respond within milliseconds and exhibit large, field-dependent changes in apparent stiffness. They also enable continuous control of damping or stiffness within a compact volume. Because of these properties, rheological materials are suitable for biomedical devices and human-machine interaction systems, where fast response and precise mechanical control are required. Compared with phase-change materials, rheological systems enable faster response and continuous stiffness control, although their achievable structural stiffness and load-bearing capability are often lower.

#### 3.2.6 Electrorheological materials

Electrorheological (ER) materials can change viscosity and yield stress quickly and reversibly when an electric field is applied [136]. This property makes them suitable for compact variable-stiffness interfaces controlled by electricity. Typical ER fluids contain polarizable particles, such as barium titanate, silica, or starch, dispersed in insulating oils at volume fractions of about 20-40%. When electric fields of 1-5 kV mm^−1^ are applied, the particles polarize and form chain-like structures along the field direction. This process generates yield stresses of up to about 10 kPa within milliseconds. Because of this fast response, ER materials can provide programmable damping, adjustable compliance, and high-bandwidth haptic feedback in small biomedical and wearable devices.

Early biomedical studies on ER materials focused on tactile display systems that create controllable surface patterns for human perception. Interest in ER-based actuation later increased with the demand for realistic haptic feedback in MIS. Han et al. [137]. integrated ER actuators into a surgical training system and enabled real-time control of force feedback during manipulation. Other studies used ER-based spherical and linear actuators to build multi-degree-of-freedom haptic interfaces with high force update rates and low mechanical inertia [120,138] (Fig. 5f). ER materials have also been studied in rehabilitation devices. Nikitczuk et al. [121]. developed a portable knee rehabilitation brace that uses ER brakes to provide task-dependent damping for motor recovery, as shown in Fig. 5g. These examples show that ER materials are effective in compact biomedical devices that require fast response and fine force control.

However, several limitations restrict the wider use of ER materials in biomedical systems. First, the maximum yield stress is relatively low, usually below 10 kPa. This limits load-bearing capacity and prevents use in applications that require high torque or structural stiffness. Second, ER fluids require high electric fields, typically 1-5 kV mm^−1^. This requirement complicates miniaturization and raises safety concerns for devices used near patients. ER performance is also sensitive to temperature and decreases as temperatures approach about 40 °C, which is close to the safety limit for skin and soft tissue. In addition, particle sedimentation can cause long-term drift in mechanical response. The use of synthetic oils and non-bioinert particles further raises concerns about long-term biocompatibility. Because of these constraints, ER materials are mainly used in low-load systems that are externally driven, such as benchtop haptic interfaces. They are less suitable for implants or devices that directly contact the patient. Without advances in low-voltage operation and biocompatible ER formulations, the application range of ER materials is likely to remain limited.

#### 3.2.7 Magnetorheological materials

Magnetorheological (MR) materials can rapidly and reversibly change their rheological behavior when a magnetic field is applied. Compared with ER fluids, MR fluids produce much higher field-induced yield stress, often one to two orders of magnitude larger. This advantage allows MR systems to deliver higher force and torque and to operate over a wider temperature range, which is important for many medical devices [139]. Typical MR fluids contain ferromagnetic or iron-based particles with sizes of about 3-5 μm dispersed in carrier media such as silicone oil, grease, elastomers, foams, or polymer gels [140]. When a magnetic field is applied, the particles align along magnetic field lines and form chain-like structures. This process enables response times below 10 ms under relatively low energy input and allows continuous control of stiffness, damping, and output torque. Because of these properties, MR materials are increasingly used in medical devices and wearable systems that require compact design, fast response, and high force output [141].

MR-based variable damping systems are widely used in lower-limb prostheses. These systems provide continuous and controllable torque, which supports smoother and more natural gait patterns [[142], [143], [144], [145]]. Typical MR ankle and knee prostheses show high energy efficiency and millisecond-level response times. They can also adapt to different walking speeds and terrain conditions. For example, the robotic ankle-foot prosthesis shown in Fig. 5h uses a torque-limited MR damper to reproduce the sagittal-plane mechanics of the human ankle joint [122]. MR fluids are also used in wearable exoskeletons for both lower- and upper-limb assistance [[146], [147], [148]]. These systems respond in real time to the user's movement intent and adjust joint torque to redistribute loads. This adjustment helps reduce muscle fatigue during motion. Fig. 5i shows an ankle exoskeleton that uses two MR clutches to control plantarflexion torque. The system supports multiple movement modes, including walking, jumping, and landing [123].

MR materials are also used in orthotic braces for knee and ankle stabilization [[149], [150], [151]]. They are further applied in rehabilitation systems for motor recovery after stroke or injury, where devices must provide precise and controllable resistance [152,153]. In recent years, MR-based actuators have gained attention in haptic interfaces and tactile displays because they can generate accurate force feedback [[154], [155], [156], [157]]. Park et al. [124]. developed a spherical MR haptic sensor with an optimized magnetic circuit, as shown in Fig. 5j. This sensor can reproduce the deformation behavior of soft tissues under dynamic loading. Such capability is useful for minimally invasive surgery, where realistic and adjustable haptic cues can improve surgical perception and training quality.

Compared with ER fluids, MR materials can generate much higher yield stress and load capacity, but they typically require larger magnetic circuits and more complex thermal management. Despite these advantages, several factors still limit the wider use of MR materials in biomedical systems. Large changes in viscosity or yield stress usually require strong magnetic fields. This requirement leads to the use of coils, magnetic cores, and thermal management components. As a result, device size and mass increase, which makes integration difficult in slender continuum robots or MIS instruments. In addition, sufficient torque output often needs a certain volume of MR fluid. This requirement further restricts compact device design. Long-term stability is another concern for MR-based systems. Magnetic particles can settle over time, and the carrier medium can age. These effects cause gradual changes in mechanical performance. At the same time, heat generated by energized coils can raise local temperatures and create risks for tissue safety. Ferromagnetic particle suspensions also raise biocompatibility concerns, which limits their use near implants or in in vivo settings. For these reasons, MR clutches and dampers are mainly used in lower-limb prostheses and exoskeletons, where larger device volumes, external power supplies, and active cooling are acceptable. By contrast, their use in slender MIS instruments or long-term implantable devices remains limited because these applications impose strict constraints on size, temperature, and sterilization. Overall, MR materials provide high load capacity and fast, controllable stiffness modulation. However, their use in medical devices requires careful balance between magnetic performance, device size, thermal safety, and long-term stability. Progress in low-power magnetic circuit design, magnetorheological elastomers, improved particle stabilization, and compact magnetic field generation may help reduce these constraints and broaden future clinical use.

#### 3.2.8 Other stimuli-responsive materials

In addition to the main categories discussed above, several other smart material systems have been explored for tunable stiffness in biomedical applications. These systems include fluid-driven compliant composites, electroadhesion-based interfaces, solvent-responsive materials, and hydrogel-based systems. Compared with phase-change and rheological materials, these approaches rely on different physicochemical mechanisms to regulate stiffness and are often more adaptable to physiological environments. In solvent-responsive systems, stiffness modulation is typically achieved through swelling-induced jamming or changes in crosslinking density under solvent exposure [[158], [159], [160]]. These mechanisms allow materials to adjust their mechanical properties in response to local chemical conditions. Electroadhesion-based systems, on the other hand, regulate interfacial forces through applied electric fields, enabling reversible transitions between compliant and stiff states without requiring bulk phase transitions. Because these approaches do not rely on large structural changes, they are attractive for compact and low-power biomedical devices. However, their load-bearing capacity and long-term reliability are still limited and require further investigation.

Hydrogel-based systems represent a more established class of biomaterials and have been widely studied in tissue engineering, drug delivery, and bio-integrated devices. Due to their high-water content and structural similarity to native extracellular matrices, hydrogels exhibit excellent biocompatibility and tissue-like mechanical behavior [[161], [162], [163]]. Their stiffness can be tuned through multiple mechanisms, including osmotic swelling, reversible chemical or physical crosslinking, ionic interactions, and network restructuring under changes in temperature, pH, light, or ionic concentration. These characteristics make hydrogels particularly suitable for applications that require mechanical compatibility with soft tissues, such as implantable devices, soft actuators, and flexible bioelectronics [164,165]. In addition, recent developments in stimuli-responsive hydrogels have enabled more precise and programmable control of mechanical properties, further expanding their functional capabilities in biomedical systems. Despite these advantages, hydrogel-based systems generally provide lower load-bearing capacity and slower response compared with phase-change or rheological materials. Their performance can also be affected by dehydration, environmental fluctuations, and long-term structural stability. As a result, hydrogels are more suitable for applications where biocompatibility and compliance are prioritized, but less effective in situations that require high stiffness or rapid mechanical switching.

Overall, these alternative smart material systems broaden the available design space for variable stiffness materials by enabling multi-stimuli responsiveness and improved environmental adaptability. However, apart from hydrogels, most of these approaches are still at an early stage of development. Further progress will require improvements in mechanical robustness, response speed, and long-term stability, as well as better integration into biomedical devices operating under realistic physiological conditions.

### 3.3 Antagonistic actuation

Antagonistic actuation controls stiffness by generating opposing internal forces within a structure. In these systems, stiffness modulation arises from the mechanical interaction and force balancing between antagonistic elements. Unlike jamming or phase-change systems, antagonistic mechanisms do not rely on external confinement or temperature-driven transitions. Instead, they regulate stiffness by applying controlled tensile, compressive, or pressure loads to structural elements such as tendons, cables, or fluidic chambers. When these opposing forces are balanced, the structure remains compliant and easy to deform. As the internal forces increase and become locked, the structure transitions into a stiffer load-bearing configuration. At the structural level, this mechanism directly addresses the long-standing soft-rigid trade-off in medical devices. In minimally invasive surgery, endoscopic tools, catheter navigation, and wearable rehabilitation systems, devices must remain soft during insertion and interaction with fragile tissue. After positioning, they must become stiff to apply force, maintain shape, or resist disturbances. Antagonistic designs meet these requirements with fast response, reversible stiffness control, and strong compatibility with slender or hollow structures. These features are difficult to achieve with purely thermal phase-change systems or confinement-based jamming approaches.

Antagonistic stiffness regulation can be implemented using three main approaches: tendon-based mechanisms, fluid-based mechanisms, and hybrid tendon-fluid mechanisms. Each approach shows different strengths and limitations in force output, control accuracy, response speed, and structural size. By using these antagonistic strategies, designers can extend the range of variable-stiffness medical robots. These mechanisms support compact structures, low energy consumption, compatibility with magnetic resonance imaging, and safe operation near patients.

#### 3.3.1 Tendon antagonism

Tendon-based antagonistic mechanisms control stiffness by applying tensile forces through cables or artificial tendons. These tendons are usually made from high-strength polymers such as Kevlar or nylon, or they are routed through soft elastomeric channels. When opposing tensile forces are applied, the structure can shift smoothly between soft and stiff states. This behavior is similar to the coordinated action of agonist and antagonist muscles in biological systems. In these systems, motors and actuators are often placed away from the deformable structure. This layout allows accurate control of shape and stiffness without adding heavy components to the device itself. As a result, tendon-based antagonistic designs reduce distal mass and improve safety. These features make them well suited for slender biomedical devices such as continuum robots.

**Fig. 6:**
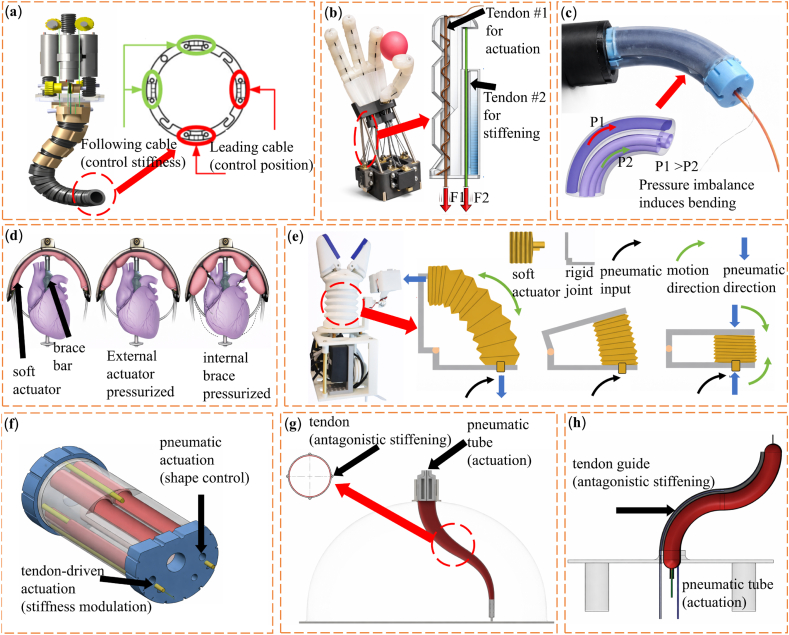
(a) Manipulator design of the actuation unit and the sensing unit [170]. (b) Anthropomorphic soft robotic gripper driven by twisted string actuators (TSAs) [171]. (c) Pneumatically actuated two-degree-of-freedom (2-DOF) bending segment for flexible surgical manipulators [172]. (d) Soft robotic device for left ventricular (LV) support [173]. (e) Tendon-driven soft robotic hand and hybrid gripper illustrating coordinated actuation mechanisms [174]. (f) Schematic of a pneumatic-tendon antagonistic actuation system enabling simultaneous deformation and stiffness modulation [66]. (g) Continuum soft manipulator combining pneumatic actuation with tendon-based antagonistic stiffness modulation [65]. (h) Inflatable soft manipulator with embedded tendons for reversible shape change and stiffness modulation [64].

In these systems, external motors drive tendons located near the base of the device. This setup allows precise control of curvature, bending direction, and overall stiffness in multi-segment continuum manipulators. Because the actuators are placed outside the instrument body, the size and mass of distal components are reduced. This design is well suited for continuum surgical robots, which must remain compact and compatible with minimally invasive deployment. In MIS, instruments must pass through narrow access channels while still generating sufficient force. Remote tendon actuation meets these requirements and is therefore widely used [[166], [167], [168], [169]]. Several MIS systems use articulated-continuum hybrid designs. These systems combine articulated rigid segments with compliant elements to enable both flexible navigation and subsequent structural stiffening. In this design, the manipulator is initially compliant to facilitate insertion and positioning, where leading cables actively drive bending while following cables provide geometric constraint. It is then mechanically stiffened through structural constraint and load-sharing once deployed, thereby improving positional stability during implant delivery, as shown in Fig. 6a. [170]. Tendon-driven mechanisms are also common in platforms for natural orifice transluminal endoscopic surgery (NOTES). In these cases, slender robotic arms must navigate long and highly curved anatomical paths [175,176]. Applications include gastrointestinal endoscopy, neurosurgery, submucosal dissection, and controlled needle insertion. Together, these examples show that tendon-based antagonistic mechanisms are effective for clinical tasks that require precise navigation in confined spaces [[177], [178], [179], [180], [181]].

Tendon-based antagonistic control is inspired by the biomechanics of the human hand and fingers. It is widely used in prosthetic hands, exoskeletons, and surgical grippers to balance flexibility and load-bearing ability in a way similar to human fingers [[182], [183], [184]]. Twisted string actuators (TSAs) are a representative implementation, offering high contraction ratios within a compact form factor. In soft robotic grippers, stiffness can be tuned via antagonistic TSA pairs, where co-contraction of opposing tendons increases internal tension without altering finger posture, thereby enhancing joint stiffness as shown in Fig. 6b [171]. Other systems use highly redundant manipulators with shape-locking linkages. In these designs, stiffness changes continuously during motion through direct control of tendon tension. Compressible tendon-driven continuum robots have also shown stiffness tuning ranges of up to about 50 times by coordinating antagonistic loading [185]. Tendon tension control has also been applied to steerable minimally invasive puncture needles. In these devices, tendon actuation enables switching between flexible and stiff states. This capability improves targeting accuracy and operational safety during needle insertion procedures [186].

Overall, tendon-based antagonistic mechanisms allow precise stiffness control in compact structures. However, these systems still face several limitations. Tendons can suffer from friction and wear during long-term use. High tension requirements also place constraints on motor size and power. In addition, nonlinear hysteresis increases control complexity [[187], [188], [189]].

#### 3.3.2 Fluidic antagonism

Fluid-based antagonistic mechanisms control stiffness by applying opposing pressures inside pneumatic or hydraulic chambers. This method allows reversible switching between soft and stiff states. It is widely used in soft robotics, where fluid-filled chambers are pressurized or depressurized in an antagonistic manner to adjust stiffness [[190], [191], [192], [193], [194]]. When multiple chambers are controlled independently, reinforced elastomer actuators can produce bending or extension in different directions. These structures form the basis of many continuum robots. They allow smooth shape changes and enable use as steerable tools in MIS [195].

One representative example in this field is the Stiffness Controllable Flexible & Learnable Manipulator for Surgical Operations (STIFF-FLOP) project. This project was inspired by the structure of octopus tentacles and focused on soft continuum manipulators for surgical use. The system introduced pneumatically driven bending modules with two degrees of freedom. These modules provided a balance between structural compliance and controllability in soft surgical manipulators, as shown in Fig. 6c [172,196,197]. Subsequent developments refined the geometry [[198], [199], [200]], reinforcement layout [201], and bending kinematics to improve dexterity and load capacity in constrained surgical environments [202,203]. Fluid-based antagonistic systems also allow additional functions through integrated fluidic channels. These functions include fluid jetting for biofilm removal [204], drug delivery using flexible reservoirs [205], and tool handling for in situ three-dimensional bioprinting [206]. Researchers have also explored fluid-driven soft actuators for tissue repair. In these cases, two-dimensional and three-dimensional shape control enables minimally invasive or noninvasive placement and deployment inside the body [207].

Pneumatically actuated origami-based structures have been used to build deployable soft retractors that can actively expand inside the cranial cavity [208]. Fluid-based antagonistic mechanisms are also well suited for cardiac assist devices. These systems are lightweight and can generate sufficient force while remaining compliant. For instance, Roche et al. [209]. developed an implantable soft sleeve that supports left ventricular contraction. The device applies synchronized circumferential compression using oriented fluidic channels. More recently, Saeed et al. [173]. proposed a pneumatically driven soft robotic ventricular assist device that augments cardiac function through coordinated actuation and structural constraint. The system consists of fluidic soft actuators positioned on the ventricular free wall and a septal bracing structure that provides an internal mechanical anchor. Upon pressurization, the actuators generate compressive forces on the ventricular wall, which are resisted by the septal brace, forming an antagonistic loading configuration. This interaction effectively increases the structural stiffness of the ventricular chamber and enhances systolic contraction, thereby restoring pulsatile blood flow and reshaping the mitral valve geometry, as shown in Fig. 6d. Similar fluid-based antagonistic designs have also been applied to assist diaphragmatic motion during inspiratory therapy to support respiratory function [210].

Overall, fluid-based antagonistic mechanisms provide smooth stiffness control in lightweight and compact structures. However, several factors still limit their use. These include relatively low force output, nonlinear pressure-volume behavior, and difficulty in maintaining accurate pressure control during fast surgical procedures.

#### 3.3.3 Tendon-fluidic hybrid antagonism

Hybrid antagonistic mechanisms combine tendon-driven and fluid-driven actuation within a single continuum structure. This combination uses the strengths of both approaches to achieve a wider range of stiffness control and improved overall performance. By applying different forms of opposing loads, hybrid systems can offer better stiffness, tunability, motion control, and adaptability to changing environments. These features make them suitable for robotic and biomedical devices. However, practical implementation remains challenging because two actuation methods must be integrated into compact medical devices with limited internal space.

One representative design integrates a modified Yoshimura origami structure with pneumatic chambers. This system enables controlled deployment and stiffness adjustment and provides a safe and low-cost option for assistive robotic devices [211]. Another common approach uses tendon-bellows hybrid structures. Zhou et al. [174]. developed a remotely operated oropharyngeal swab system that combines a soft robotic hand with a flexible wrist. The system supports single-use swab grasping and manipulation with dexterity close to that of the human hand. Pneumatic actuation controls joint motion and gripping force, while tendons fine-tune grasp shape and stiffness, as shown in Fig. 6e. Similar hybrid concepts have been applied to continuum manipulators for MIS. Mathur et al. [66]. reported a silicone-based continuum manipulator integrating pneumatic actuation with tendon-based stiffening. While pneumatic chambers enable compliant bending, the tensioned tendons constrain deformation along the manipulator body. This interaction can be interpreted as an antagonistic mechanism, where internal pressure and tendon tension collectively contribute to an increase in effective structural stiffness. Luo et al. [212]. proposed a distal joint that combines pneumatic chambers with cable actuation. This design shows stable bending behavior in both soft and stiff states and maintains consistent motion during stiffness switching. It also meets the load requirements of gastrointestinal endoscopic robots. Other studies have presented soft manipulators that integrate pneumatic actuation with tendons, as shown in Fig. 6g and h [64,65]. In these systems, internal pressurization drives expansion and shape change, while embedded tendons provide tensile constraints that oppose deformation, enabling tunable stiffness through antagonistic interaction. By coordinating pressure and tendon tension, the manipulators can transition between a highly compliant, compact state and a more rigid, deployed configuration. This reversible transformation is particularly advantageous for MIS, where devices must navigate confined anatomical pathways and subsequently provide sufficient structural support and positional stability at the target site.

In summary, tendon-based antagonistic mechanisms offer high control accuracy and good performance in fine manipulation tasks. However, they often require relatively large actuators and may suffer from reliability issues such as tendon wear. Fluid-based antagonistic mechanisms can generate higher forces and provide smooth actuation in compact structures. Their performance, however, depends strongly on accurate pressure control. Tendon-fluid hybrid mechanisms further improve compliance and control precision, but they also increase mechanical complexity and control difficulty. These factors raise system cost and make integration more challenging. From the perspective of clinical translation, tendon-based antagonistic mechanisms are currently the most mature option for minimally invasive surgery and endoscopic systems. In contrast, purely fluid-based and hybrid antagonistic approaches are still limited by bulky pneumatic components, lower energy efficiency, and complex control requirements. These limitations make large-scale clinical adoption difficult in the near term.

### 3.4 Cross-mechanism comparison

**Fig. 7:**
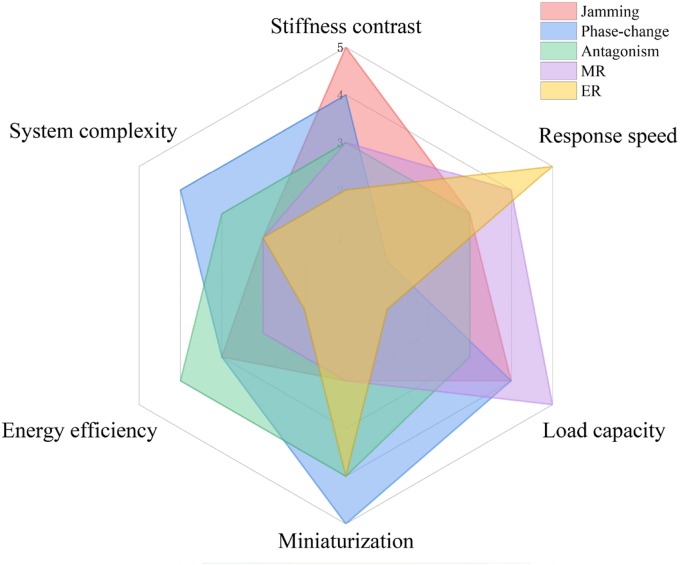
Scores represent normalized qualitative assessments derived from performance ranges reported in literature across stiffness contrast, response speed, load capacity, miniaturization potential, and system-level complexity.

To provide a systematic overview of variable stiffness strategies, Table 1 summarizes representative systems and materials, actuation mechanisms, fabrication methods, biocompatibility, and achievable stiffness/force ranges, together with their advantages and limitations, while Fig. 7 presents a normalized comparison across key performance metrics. The qualitative scores (1-5) in Fig. 7 are derived from representative device-level performance ranges reported in the literature (Table 1). For stiffness contrast, scores range from minimal modulation (<1.5 × , score 1) to large contrast (>25 × , score 5). Response speed spans from millisecond-scale actuation (<0.1 s, score 5) to multi-second thermal transitions (>30 s, score 1). Similar interval-based criteria are applied to load capacity, miniaturization feasibility, and system complexity, with thresholds defined consistently across mechanisms to ensure comparability under unified benchmarks. This comparison highlights system-level performance differences and key engineering trade-offs among the mechanisms.

**Table 1:** Comparison of representative variable-stiffness mechanisms based on jamming, phase-change, and antagonistic strategies.

Mechanism	Material system	Activation/Stimulus	Fabrication	Biocompatibility	Tunable Stiffness/Force Range	Key Advantages/Limitations	Ref
Jamming (Granular jamming)	3D-printed interlocking particles (PA12)	Negative pressure	MJF 3D printing	Not reported	Stiffness increase: >25 ×	Adv.: Flexible when unjammed; highly stiff when jammed; shape-adaptive. Lim.: Requires sealed enclosure; hysteresis; structural complexity.	68
Jamming (Granular jamming)	Dual-layer 3D re-entrant cells (polymer structures)	Negative pressure	3D printing	Not reported	Stiffness increase: up to 67%	Adv.: Wide stiffness range; lightweight; 3D-printable. Lim.: Needs vacuum setup; hysteresis and wear.	77
Jamming (Programmable tile-scale locking)	SAILS programmable interlocking tiles (TPU)	Negative pressure	FDM 3D printing	Not reported	Stiffness increase: up to 3 ×	Adv.: Morphing shape & stiffness; large contrast. Lim.: Requires sealed vacuum; geometry-dependent response.	78
Jamming (Granular jamming)	Dual-faced interlocked structures (PA12)	Negative pressure	MJF 3D printing	Not reported	Stiffness increase: 30-40 ×	Adv.: High specific strength, energy absorption, impact resistance, and dual-faced tunable bending. Lim.: Requires air supply; performance depends on geometry and sealing.	84
Jamming (Granular jamming)	Folded-plate variable-stiffness soft gripper; silicone outer layers, particles and FPM	Negative pressure	Silicone casting and molding	Not reported	Stiffness increase: 3 ×	Adv.: Multidirectional grasping with tunable stiffness; enhanced load capacity. Lim.: Requires vacuum hardware; structurally complex; prone to wear and hysteresis.	81
Jamming (Layer jamming)	Foldable layer-jamming soft retractor (taffeta fabric, polycarbonate layers and silicone friction surface)	Negative pressure	Heat sealing, laser cutting, and layered assembly	In-porcine validated	Stiffness increase: 3-5 ×	Adv.: Minimally invasive; reversible stiffening. Lim.: Requires airtight seal; sensitive to layer design.	83
Smart material (Phase-change alloy)	Bi/In/Sn/Zn low-melting-point alloy cement	Thermal	Alloy melting, mixing and injectable molding	Good biocompatibility; low cytotoxicity; in vivo validated	Compressive strength: 37.6 MPa; bending modulus: 6.41 GPa; reversible liquid–solid transition	Adv.: Rapid molding; re-meltable; reusable. Lim.: Requires heating; compressive/bending strength lower than PMMA standard; limited load-bearing use.	115
Smart material (Phase-change alloy)	LESS robotic manipulation arm with articulated section and CSCS filled with PCM alloy (Ga/In/Bi/Cu)	Thermal	Mechanical assembly with PCM filling and integrated water-circulation/heating	Not reported	Stiffness increase: 3.03–4.12 × (arm); maximum payload: 10 N	Adv.: Switchable compliance; high payload. Lim.: Thermal control needed; slow cooling.	116
Hybrid (SMA and tendon antagonism)	Spring-based continuum robot (MINIR-II) with SMA spring backbone and tendon routing	Thermal and tendon tension	3D printing and tendon-driven assembly	MRI-compatible	Stiffness increase: up to 2 ×	Adv.: Segment-wise tuning; MRI-safe. Lim.: Slow thermal response; modest stiffness gain.	117
Smart material (phase-change polymer, SMP)	Conductive shape memory polymer (CSMP)-based magnetic catheter (VST)	Thermal	Dipping-based multilayer coating (CSMP, PTFE, silicone)	Cytocompatible (ISO 10993 tested)	Stiffness change factor ≈ 21; modulus drop ∼20 × (2 → 0.1 GPa)	Adv.: Integrated sensing/heating/stiffness; miniaturizable (2 mm); biocompatible. Lim.: Slow response (seconds), thermal control needed	119
Smart material (SMA-based sheath)	Continuum manipulator with SMA woven sheath, SMA core, silicone shell, ABS connectors, Dyneema cables	Thermal	Woven SMA sheath fabrication and modular assembly	Not reported	Stiffness increase: up to 223.1% (sheath); 139.2% (module)	Adv.: Continuous stiffness tuning; relatively fast response; compact and suitable for miniaturization. Lim.: Thermal hysteresis; temperature-dependent performance; reduced module-level gain.	213
Smart material (magnetorheological)	MR clutch-powered ankle exoskeleton with hydrostatic transmission	Magnetic field	Mechanical integration of MR clutches, motor, gearbox, and hydraulic system	Not reported	Torque range: up to 90 N m	Adv.: High torque output; multifunctional locomotion support. Lim.: Bulky and tethered; requires parameter tuning.	123
Antagonism (Tendon antagonism)	Cable-driven rigid–flexible links with coil-spring backbone	Cable tension	Metal sintering (Ti6Al4V) and mechanical assembly	Not reported	Stiffness increase: 1 × -9 ×	Adv.: Compact design; tunable stiffness; suitable for implant delivery. Lim.: Requires pose-specific adjustment; limited stiffness range.	170
Hybrid antagonism (Pneumatic and tendon/cable-driven)	Antagonistic SMA springs and SCP strings in silicone continuum manipulator	Thermal	Silicone molding and actuator integration	Not reported	Stiffness increase: up to 2.6 × ; loading capacity: up to 4.8 N	Adv.: Reduced hysteresis; effective bending in both states; suitable for grasping and monitoring. Lim.: Thermal switching limitations; relatively large diameter.	214
Hybrid (Phase-change and tendon antagonism)	Helical spring-like continuum joint, Nitinol backbone and flat TPU air tubes	Positive pressure and tendon/cable actuation	MJF 3D printing, extrusion and assembly	Not reported	Stiffness increase: ≈3.2 × ; loading capacity: 1.5-4.8 N	Adv.: Fast transition (<3 s); shape-locking; improved load capacity. Lim.: Requires pneumatic source; moderate stiffness ratio; relatively large size.	212
Hybrid (SMA and tendon antagonism)	Tendon-driven continuum robot with embedded phase-change material	Thermal and tendon antagonism	Mechanical assembly with embedded PCM and heating elements	Not reported	Not quantitatively reported	Adv.: Wide tunable stiffness; stiffness adjustable without position change. Lim.: Slow thermal response; antagonistic torsion weakens PCM effect.	215
Antagonism (Tendon antagonism)	Underactuated antagonistic tendon-driven continuum robot (OctRobot-I)	Antagonistic tendon tension	Custom continuum robot platform	Not reported	Antagonistic tendon force: μ = 0-45 N tested	Stiffness tunable via antagonistic tendon force; open-loop tendon force μ: 0-45 N tested	216

However, beyond intrinsic mechanical performance, fabrication and packaging constraints fundamentally limit the achievable device size and feature resolution of each approach. For architected jamming systems that rely on printed microstructures and sealed membranes, geometric fidelity at the sub-millimeter scale is often a practical limit: PolyJet-based characterization of microstructures shows that achieving the designed feature height requires a minimum designed width of ≈300 μm for 16-28 μm layer thickness modes, and ≈500 μm for 36 μm layers, highlighting an intrinsic resolution threshold for reliable microfeature formation [217] For polymer-based components fabricated by FDM, mechanical performance is sensitive to layer height within commonly used settings; PLA tensile testing across 0.05-0.30 mm layer heights shows measurable strength variation, indicating that print settings can directly influence load-bearing repeatability of compliant backbones used in antagonistic structures [218]. In contrast, phase-change materials approaches have demonstrated strong potential for miniaturization, particularly in catheter-scale systems where stiffness modulation is achieved through intrinsic material transitions. For example, a submillimeter continuous variable-stiffness catheter with a 1 mm outer diameter and a 180 μm working channel integrates a low-melting-point alloy core while maintaining a surface temperature below 43 °C through encapsulation design. This example highlights that, at small scales, system performance is no longer governed solely by intrinsic material behavior, but is increasingly constrained by thermal transport pathways, encapsulation thickness, and heat dissipation efficiency [59]. More broadly, a wide range of phase-change materials have been implemented using additive manufacturing techniques for biomedical applications. Under such material-driven stiffness regulation mechanisms, the mechanical performance of printed structures becomes highly sensitive to fabrication parameters, including printing resolution, interlayer bonding, and process-induced anisotropy [[219], [220], [221]]. To systematically capture these effects, Table 2 compares representative additively manufactured variable-stiffness systems, highlighting how material systems and printing methods collectively influence structural resolution, mechanical performance, and their suitability for different biomedical applications, together with the associated advantages and limitations.

**Table 2:** Additive manufacturing of variable-stiffness materials for biomedical applications: materials, methods, and performance comparison.

Material system	Printing method	Resolution	Potential biomedical applications	Advantages	Limitations	Ref
Shape-memory polymers	SLA/DIP	High (typically 10-50 μm)	Minimally invasive devices; drug delivery systems	High resolution; excellent surface quality; complex microstructures	Limited material options; restricted to photocurable SMPs; difficult multimaterial integration	[[222], [223], [224], [225], [226]]
	FDM	Moderate (typically 100-300 μm)	Tissue scaffolds; orthopedic implants; biomedical prototypes	Low cost; process simplicity; thermoplastic compatibility	Low resolution; poor surface finish; limited geometric complexity; weak multimaterial capability	[[227], [228], [229]]
	DIW	Moderate to low (typically 100 μm–1 mm)	Soft robotics, wearable biomedical devices; bio-integrated actuators	Multimaterial capability; functional integration; material versatility	Limited resolution; rheology-dependent process; structural instability during printing	[230,231]
Low-melting-point alloys	FDM	Moderate (typically 100-500 μm)	Flexible electronics; wearable biomedical devices; soft sensing systems	Simple processing; scalable fabrication; hybrid compatibility	Limited precision; flow control challenges; process modification required	[228,[232], [233], [234], [235]]
	DIW	Moderate to high (10-200 μm)	Soft bioelectronics; wearable sensors; implantable devices	Direct ink writing of liquid metals; conformal deposition; high electrical conductivity	Oxidation-induced instability; limited structural stability; rheology dependence	[[236], [237], [238], [239], [240], [241], [242], [243]]
	Liquid-phase 3D Printing	Moderate (100-500 μm)	Microfluidics; implantable electronics; conductive architectures	Enables low-viscosity metal printing; rapid solidification	Process complexity; limited resolution; liquid-environment constraints	[244]
Thermoplastic polymers	FDM	Moderate (typically 100-300 μm)	Tissue scaffolds; patient-specific implants; surgical guides; biomedical prototypes	Low cost; process simplicity; broad thermoplastic compatibility; good structural control	Limited surface quality; relatively low resolution; thermal degradation risk; restricted multimaterial capability	[[245], [246], [247], [248]]
	DIW	Moderate (typically 100-400 μm, nozzle-dependent)	Tissue scaffolds; drug-delivery constructs; bio-integrated polymer architectures	Mild processing conditions; suitable for solvent-based thermoplastic inks; compositional flexibility	Rheology-sensitive process; reduced shape fidelity compared with photopolymerization; post-drying effects; limited build speed	[248,249]
	SLS	Moderate to high (typically 60-150 μm)	Bone scaffolds; bioactive implants; drug-delivery devices	No support structure required; complex porous architectures; suitable for thermoplastic powders	Powder preparation is demanding; thermal shrinkage/warpage; limited material palette; inherent surface roughness	[[250], [251], [252]]
Hydrogels	SLA/DLP	High (typically 10-100 μm)	Tissue engineering scaffolds; microfluidic devices; drug delivery systems	High resolution; excellent structural fidelity; complex architectures	Limited to photocurable hydrogels; potential cytotoxicity of photoinitiators; material constraints	[[253], [254], [255]]
	DIW	Moderate (typically 100-500 μm)	Tissue scaffolds; biofabrication; cell-laden constructs; soft biomedical devices	Cell-friendly processing; multimaterial capability; broad bioink compatibility	Limited resolution; rheology-dependent; structural instability during printing	[[256], [257], [258], [259]]
	Inkjet bioprinting	High (typically 20-100 μm, droplet-based)	Drug delivery; cell patterning; tissue models	High speed; precise droplet control; low material consumption	Limited viscosity range; nozzle clogging; limited mechanical integrity of printed constructs	[260,261]

A cross-mechanism comparison shows that jamming, phase-change, rheological, and antagonistic strategies occupy distinct regions of the variable-stiffness design space. However, superiority is inherently constraint dependent. When large stiffness contrast and shape locking are prioritized over miniaturization and untethered operation, jamming architectures are advantageous. In thermally controlled and compact systems where intrinsic material transitions are feasible, smart material-based approaches offer high stiffness density but remain limited by cooling dynamics and encapsulation durability. Rheological systems are preferable in applications demanding sub-second response and continuous tunability, yet their relatively low yield stress density and packaging overhead reduce suitability for implantable or highly miniaturized devices. Antagonistic actuation integrates naturally with continuum robotic structures and allows distributed stiffness modulation, but shifts the performance bottleneck toward actuator efficiency, fatigue resistance, and control robustness rather than intrinsic material capability.

Overall, no single stiffness regulation mechanism dominates across all criteria. The practical performance of each approach is not solely determined by stiffness modulation capability but is also constrained by system-level factors such as packaging reliability, thermal management, and long-term mechanical stability. Mechanisms that balance moderate stiffness contrast with structural simplicity, scalable fabrication, and integration feasibility are therefore more advantageous for real-world implementation than those maximizing stiffness modulation at the expense of system complexity. These considerations explain the increasing emergence of hybrid strategies and highlight the importance of mechanism-application co-optimization rather than isolated material comparison.

## 4 Applications

Variable stiffness mechanisms are now used in many biomedical systems. These mechanisms allow devices to adjust their mechanical behavior in response to anatomy, surgical tasks, and patient-specific needs. Based on the mechanism-level analysis in Section 3, this section reviews how jamming-based systems, smart material-based systems, antagonistic actuation, and hybrid strategies are applied in five biomedical areas. These areas include drug delivery devices, wearable assistive devices, MIS, adaptive sensing systems, and bone repair and orthopedic applications.

### 4.1 Drug delivery devices

Modern drug delivery systems increasingly require devices to remain soft and compliant during navigation, while exhibiting sufficient mechanical stability during anchoring, retention, or on-demand release. Conventional oral or implantable carriers face significant challenges in balancing these competing requirements and often suffer from poorly controlled release profiles, low bioavailability, or insufficient mechanical adaptability to the dynamically changing gastrointestinal and vascular environments. VSMs address these limitations by enabling devices to switch between a deformable transport state and a rigid functional state, thereby improving targeting accuracy, in vivo retention time, and responsiveness to external stimuli. Among existing variable-stiffness mechanisms, phase-change materials (e.g., LMPAs, SMPs) and magnetoactive transitional systems are particularly well suited for drug delivery because they provide large stiffness contrast, allow non-contact activation, offer shape adaptability, and enable confined-space reconfiguration [262].

**Fig. 8:**
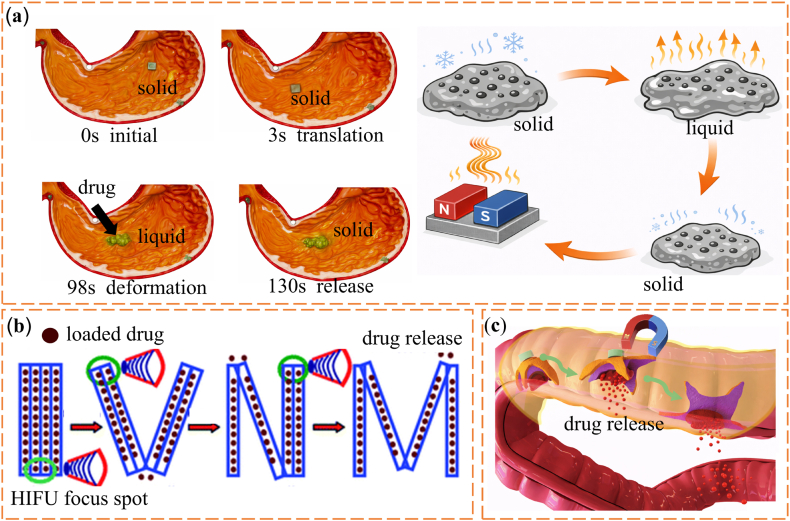
(a) MPTMs performing drug delivery [263]. (b) Schematic illustration of the HIFU-enabled spatial and temporal control of shape recovery and drug release [264]. (c) Magnetically guided multilayer carrier for targeted gastrointestinal delivery [265].

Magnetoactive phase-transitional matter (MPTM) is a promising material for mechanically adaptive oral drug delivery systems. Wang et al. [263] showed that MPTM can act as reconfigurable micro-actuators and so-called universal helices. These structures support locomotion, component assembly, and targeted drug release in confined gastric models, as shown in Fig. 8a. When an alternating magnetic field is applied, the material switches between solid and liquid states. This transition allows controlled deformation and drug release. As a result, the capsule remains mechanically robust during ingestion and becomes compliant during release or retrieval. This activation method is noninvasive and well suited for gastrointestinal applications, because external magnetic fields can penetrate tissue and allow remote control from outside the body. SMPs provide another approach for variable-stiffness drug delivery. In these systems, temperature-induced shape changes regulate drug release. Li et al. [264] reported an SMP-based delivery platform actuated by high-intensity focused ultrasound (HIFU), where localized energy deposition enables spatially selective and temporally programmable softening. The triggered shape recovery is intrinsically coupled with drug release, allowing precise spatiotemporal control over therapeutic delivery, as shown in Fig. 8b. Vakil et al. [266] proposed a magnetically responsive SMP system for implantable drug reservoirs. In this design, alternating magnetic fields enable remote control of release rate. These studies show that stiffness changes in SMPs can link shape transformation with diffusion pathways, which enables externally triggered and dosage-controlled drug delivery. In addition to phase-change materials, soft robotic carriers with tunable stiffness have been explored for targeted drug administration. Heunis et al. [265] developed a multilayer soft carrier made of PDMS, SMP layers, and magnetically driven neodymium-iron-boron disks, as shown in Fig. 8c. This carrier shows programmable shape change, controlled retention, and thermally responsive actuation. These features support precise navigation and site-specific drug delivery in gastrointestinal environments.

In summary, variable-stiffness mechanisms effectively address the long-standing trade-off between compliant navigation and stable anchoring in drug delivery systems. By enabling state switching between soft transport and rigid deployment, these mechanisms enhance positioning accuracy and release control under dynamic physiological conditions. From a translational perspective, however, not all mechanisms show equal clinical readiness. Phase-change systems based on LMPAs or SMPs are currently the most mature for gastrointestinal applications because they allow compact integration and remote activation without requiring bulky onboard hardware. In contrast, magnetoactive systems that rely on dispersed nanoparticles or complex multi-material architectures introduce additional safety and regulatory uncertainties, particularly regarding long-term material retention and thermal dose control. The principal clinical bottlenecks in this domain are not stiffness tunability itself, but rather thermal safety management, repeatable activation under variable in vivo heat dissipation, and long-term biocompatibility of encapsulation and magnetic components. Cooling delays, incomplete phase recovery, and material fatigue over repeated activation cycles remain practical constraints. Therefore, the most promising direction for near-term translation lies in simplified, thermally compliant phase-change architectures with robust encapsulation strategies, while more complex hybrid soft-robotic systems may require further validation before routine clinical deployment.

### 4.2 Wearable devices

Wearable biomedical devices must remain in close and conformal contact with the soft and highly deformable surface of the human body. At the same time, these devices often need to provide mechanical support, transmit forces, or deliver haptic feedback during use. Conventional rigid designs struggle to meet both requirements. They frequently cause motion artifacts, discomfort during wear, and reduced signal quality [267]. VSMs address this limitation by enabling adaptive mechanical behavior. Devices incorporating VSMs can stay compliant during natural body motion. When mechanical support or stabilization is required, they can stiffen in a controlled manner. This capability allows wearable systems to assist grasping, support external loads, and stabilize movement without compromising comfort.

**Fig. 9:**
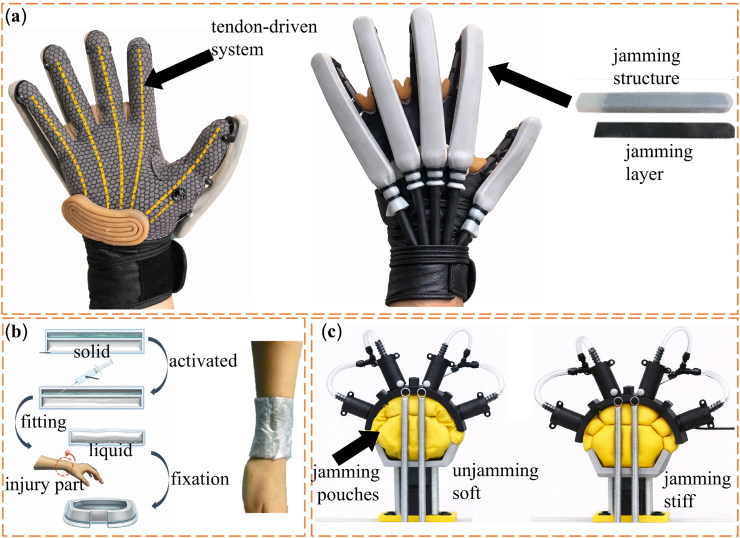
(a) The soft glove system consists of a glove, a tendon-driven system, and a pneumatic module with four soft actuators and five laminar jamming structures [268]. (b) A smart bandage designed with BiInSn and Al-NaOH-composited eGaIn [269]. (c) Granular jamming-based interface exhibiting a transition from a conformable state to a stiffened state for enhanced attachment stability [270].

Recent progress in wearable robotics illustrates this trend clearly. Exoskeleton gloves have shifted from early rigid, motor-driven designs toward lightweight systems integrated with textiles. In these newer designs, functional assistance is achieved through tunable stiffness mechanisms rather than fully rigid structures. As shown in Fig. 9a, one representative glove integrates laminar jamming layers into a soft fabric substrate to increase grasp stability by adjusting finger stiffness. Miniature actuators drive finger flexion through tendon transmission. The jamming layers then lock the finger configuration during object manipulation [268]. This combination of compliant actuation and adjustable passive stiffness improves hand dexterity and task stability while preserving wearing comfort and natural motion.

LMPAs provide another practical approach for achieving tunable stiffness in wearable systems. This approach is especially useful for jointed exoskeleton modules that require high torque and structural rigidity during specific phases of operation. When LMPA cores are embedded within polymer-based joint structures, the device can remain compliant during normal movement. Under localized heating, the alloy solidifies and the joint rapidly transitions into a load-bearing state [125,126]. These hybrid joint designs represent early engineering examples of on-demand stiffening in exoskeletons. They provide mechanical support comparable to conventional metal joints while preserving the safety and adaptability typically associated with soft robotic systems.

Beyond wearable assistive devices, variable stiffness systems based on liquid metals (LMs) have also been studied for clinical uses such as orthopedic fixation and trauma care. Yuan et al. [269]. developed an intelligent fixation bandage that combines a Bi-In-Sn alloy with an eGaIn layer and is activated through an Al-NaOH reaction, as shown in Fig. 9b. During application, the bandage remains conformable and adapts to the patient's anatomy. After activation, the liquid metal solidifies and forms a rigid, splint-like structure. Mechanical tests show that this liquid metal bandage has much higher tensile and bending stiffness than conventional fiberglass casts. At the same time, it preserves good conformity during placement. This example shows how variable stiffness mechanisms can combine personalized fitting with high mechanical stability, offering an alternative approach to fracture fixation and trauma management that balances adaptability and load-bearing performance.

Jamming-based structures are increasingly used in wearable assistive systems. These systems are well suited for applications that require fast switching between soft and stiff states to balance wearing comfort and task performance. Brignone et al. [270]. developed a wearable support device based on a granular jamming membrane, as shown in Fig. 9c. In the unjammed state, the membrane closely conforms to the body surface. When vacuum pressure is applied, the structure rapidly stiffens. This device provides adaptive support for joints or limbs while remaining lightweight and low profile. The example shows that jamming mechanisms can meet the demands of long-term wearable assistance, where comfort, stability, and simple system design are all required.

Overall, variable stiffness mechanisms allow wearable systems to balance comfort, adaptability, and load-bearing capability, which is difficult to achieve with materials of fixed stiffness. However, wearable applications impose stricter practical constraints than many other biomedical scenarios. Devices intended for daily or long-term use must remain lightweight, breathable, energy-efficient, and mechanically stable under repeated loading cycles. Under these conditions, stiffness tunability alone is not sufficient to determine translational potential. Jamming-based textiles and tendon-driven antagonistic structures rely primarily on mechanical reconfiguration and therefore align more naturally with long-term wearable requirements. In contrast, thermally activated phase-change systems require repeated heating and cooling cycles, which may introduce additional energy demands and thermal management considerations. Rheological systems offer rapid response, yet their dependence on sealed chambers and embedded field-generating components can increase system mass and reduce breathability, factors that are particularly critical for continuous wear. From a clinical perspective, the main bottleneck in wearable variable stiffness systems lies in the integration of compact actuation, durable encapsulation, and stable long-term performance within soft and lightweight architectures. Cyclic fatigue, environmental exposure, and user variability further complicate reliability. As a result, mechanically simpler and textile-compatible solutions currently appear more compatible with near-term wearable deployment, whereas more complex thermally or field-activated systems may remain better suited to intermittent or task-specific use until energy efficiency and packaging constraints are further addressed.

### 4.3 Minimally invasive surgical devices

MIS instruments must satisfy two conflicting requirements. During insertion and navigation, the instruments need sufficient compliance to move safely through narrow, curved, and delicate anatomical pathways. After reaching the target site, the same instruments must become rigid enough to transmit forces, maintain shape, and allow precise manipulation. This conflict is often referred to as the “soft-rigid paradox” and makes MIS one of the application areas with the strongest demand for tunable stiffness. For this reason, catheters, probes, and continuum surgical robots with variable stiffness have attracted increasing research attention. These systems aim to improve surgical safety, enhance manipulation accuracy, and increase overall procedural control.

**Fig. 10:**
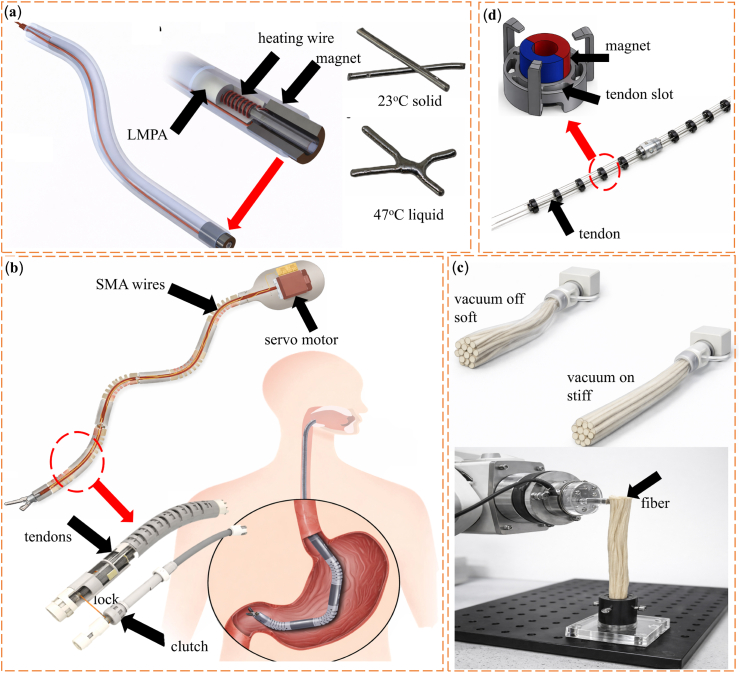
(a) Schematic views of the continuous variable stiffness (CVS) catheter [59]. (b) Schematic illustration of the proposed bioinspired time-share driven robot [272]. (c) Fiber jamming-induced stiffness transition and corresponding experimental setup [273]. (d) Magnetic locking unit and its integration within a tendon-driven continuum robot for stiffness modulation [274].

LMPAs are among the most actively explored VSMs for MIS because their solid-liquid phase transitions can be triggered near physiological temperatures, allowing catheters to switch between compliant and rigid states without bulky hardware [271]. Luss et al. [59]. developed an LMPA-filled catheter for robot-assisted epiretinal membrane peeling, as shown in Fig. 10a. When Joule heating is applied, the alloy melts and the bending stiffness of the catheter decreases. This soft state enables safe and compliant navigation. After heating is turned off, the alloy solidifies quickly and restores a stiff backbone for precise manipulation. This design enables local stiffness control along the catheter length. It allows surgeons to perform delicate microsurgical tasks while reducing mechanical stress on surrounding tissues.

A complementary design approach combines tendon-driven actuation with phase-change-based locking to build continuum robots that provide both high dexterity and tunable stiffness. Wang et al. [272]. proposed a time-shared actuation scheme in which a single motor drives multiple steerable segments in sequence. Each segment integrates SMA clutch that functions as a local locking unit, as shown in Fig. 10b. When electrical heating is applied, the SMA clutch softens and disengages. This state allows the selected segment to bend locally. After cooling, the clutch re-engages and the segment becomes stiff again, restoring its load-bearing capability. In this layered design, tendons generate motion while phase-change locking units provide stiffness and structural support. This separation of functions reduces the number of actuators and the overall hardware density. As a result, highly articulated MIS instruments can achieve efficient deployment and precise manipulation in confined access routes such as single-port or natural-orifice pathways.

Jamming-based mechanisms provide another effective way to achieve large and reversible stiffness changes in minimally invasive surgical instruments. Brancadoro et al. [273]. investigated fiber jamming as a stiffening strategy for catheter-like soft robotic structures, as shown in Fig. 10c. In this approach, bundles of internal fibers are enclosed within an external membrane, and stiffness is tuned by vacuum-induced jamming: when negative pressure is applied, the fibers are compacted, inter-fiber friction increases, and the structure transitions to a stiffer state. The stiffening performance depends strongly on the material properties and arrangement of the filling fibers, as well as on the external membrane. This mechanism provides a promising route for endoscopic devices, where high axial stiffness is required for force transmission, while compliant behavior in other directions helps reduce tissue damage. Similarly, Pogue et al. [274]. proposed a magnetic locking mechanism for tendon-driven continuum robots. In their design, an external magnetic field causes internal plates to rotate and mechanically engage with bead-like elements arranged along the tendons, as shown in Fig. 10d. This interaction increases structural stiffness without active heating. Both systems rely on passive, geometry-based constraints to regulate stiffness. As a result, they avoid thermal safety and heat dissipation issues associated with LMPAs and SMPs.

Overall, variable stiffness mechanisms have substantially expanded the design space of MIS instruments at the prototype level. They allow a single device to combine compliant navigation with localized rigidity for force transmission and precision manipulation. However, translational progress remains limited. The primary constraint is not the achievable stiffness contrast or steering dexterity, but the reliability of repeated operation under clinical processing conditions. In thermally activated systems such as LMPA-filled catheters, long-term encapsulation integrity and predictable phase-transition behavior must be maintained over repeated heating cycles [59]. Any leakage, incomplete solidification, or temperature overshoot may compromise both safety and mechanical consistency. Sterilization exposure introduces additional thermal and material stresses, which can further affect repeatability [275]. For tendon–SMA hybrids, the mechanical architecture is effective in principle, yet its performance depends on coordinated timing between actuation and clutch engagement. This increases control complexity and raises challenges in validation, calibration, and intraoperative reliability [41]. Jamming-based and magnetically locked mechanisms avoid direct thermal cycling and therefore reduce some safety concerns. Their structural logic is mechanically intuitive and often fails in a compliant rather than hazardous manner. Nevertheless, vacuum routing, pressure sealing, or magnetic components occupy space within already constrained endoscopic channels. Integration into standard instrument geometries therefore becomes a dominant engineering constraint. From a clinical standpoint, the decisive bottleneck in MIS is not stiffness magnitude but robustness, sterilization compatibility, and seamless workflow integration [276]. Mechanisms that require minimal additional hardware, tolerate repeated processing, and default to safe mechanical states are more likely to progress toward routine use. This perspective explains why mechanically steerable catheters and passive shape-locking guidewires remain prevalent in operating rooms, while more complex thermally or electrically activated variable stiffness systems are still largely confined to experimental platforms.

### 4.4 Sensors

VSMs enable new sensing strategies by allowing sensors to adjust their mechanical compliance during operation. Unlike conventional sensors, whose mechanical properties are fixed after fabrication, VSM-based sensing systems can actively change their structural state. These sensors can remain soft when high sensitivity is needed to detect weak signals. When exposed to large loads or deformations, they can stiffen to maintain structural integrity and measurement reliability. Through this adaptive behavior, variable stiffness sensors offer a practical way to reduce the trade-offs between sensitivity, dynamic range, and mechanical stability in biomedical sensing.

A key challenge in pressure sensor design is the trade-off between sensitivity and bandwidth. Sensors with highly compliant structures are usually very sensitive, but they often saturate at moderate pressure levels. In contrast, stiffer sensors can operate over a wider bandwidth and tolerate higher loads, but they respond poorly to weak physiological signals. VSMs offer a way to switch between these two operating states during use. With this capability, a single sensor can adapt its mechanical state to different measurement tasks. As a result, VSM-based sensors can support wide-range and multimodal sensing, which extends the practical capability of biomedical sensing systems.

**Fig. 11:**
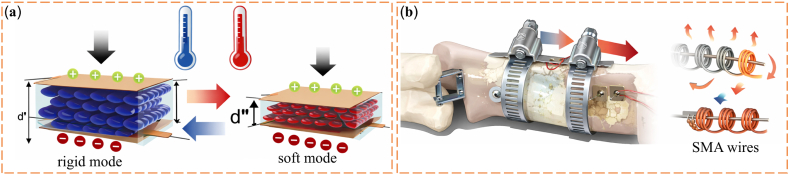
(a) Thermally induced phase transition of gallium microgranules enabling reversible switching between soft and rigid states for adaptive pressure sensing [69]. (b) SMA-actuated non-bonded piezoelectric sensing configuration enabling impedance measurement through controlled modulation of interfacial contact stiffness [278].

LMPAs can help address the sensitivity–bandwidth trade-off by allowing sensors to switch between soft and stiff states in a controlled way [277]. This behavior relies on the reversible solid–liquid phase transition of the alloy. Lee et al. [69]. developed a capacitive pressure sensor that uses gallium droplets as a phase-change dielectric layer, as shown in Fig. 11a. In the liquid state, the sensor remains highly deformable and reaches a sensitivity of up to 16.97 kPa^−1^. This high sensitivity enables reliable detection of weak physiological signals, such as pulse and respiration. After solidification, the sensor operates over a much wider pressure range. The upper pressure limit increases to about 1.45 MPa, which allows measurement under high-load conditions. Through this dual working mode, the sensing range expands from 3 Pa to 1.45 MPa. This range exceeds that of human skin and most conventional flexible pressure sensors.

SMAs provide another route to tunable behavior by combining controllable shape change with mechanically strong actuation. Srivastava et al. [278]. developed an SMA-based actuator for non-contact piezoelectric bone diagnostics, as shown in Fig. 11b. In this system, the SMA actuator provides controllable displacement and force output through thermally induced phase transformation. By adjusting the actuation level, the device regulates the contact pressure between the piezoelectric sensor and the bone surface without requiring direct bonding. This modulation of contact force effectively alters the interfacial contact stiffness, thereby stabilizing the sensing interface and improving impedance measurement accuracy. Unlike material-based stiffening approaches, this strategy achieves stiffness regulation through active control of boundary conditions. As a result, it enables reliable signal acquisition while minimizing disturbance to surrounding tissues.

Overall, variable stiffness sensing architectures expand the functional envelope of biomedical sensors by enabling context-dependent mechanical adaptation. However, the practical bottleneck in clinical translation is not merely sensing range or sensitivity, but long-term stability and packaging reliability under repeated mechanical and environmental exposure. For LMPA-based sensors, although the achievable sensing range is exceptionally broad due to solid-liquid transitions, repeated phase cycling introduces concerns regarding encapsulation integrity, alloy redistribution, and thermal management. Maintaining consistent dielectric behavior over prolonged use remains a nontrivial engineering challenge. Similarly, while SMA-assisted sensing platforms provide controllable actuation–sensing coupling, their reliance on active heating and cooling increases energy demand and control complexity, which may limit miniaturization and long-term implantable applications. In contrast, sensing strategies that achieve tunability through structural reconfiguration or passive mechanical adaptation may offer greater robustness in wearable or continuously operating systems. From a translational perspective, the most promising directions are likely those that minimize thermal cycling, reduce reliance on high-power actuation, and maintain stable calibration under physiological variability. In this sense, the core bottleneck for variable stiffness sensing is not sensitivity enhancement itself but ensuring reproducible mechanical-electrical coupling under real-world conditions.

Future opportunities lie in combining adaptive stiffness control with closed-loop robotic systems, wearable monitoring platforms, and minimally invasive diagnostic tools, where mechanical stability, low power consumption, and calibration robustness are as critical as raw sensing performance.

### 4.5 Bone repair and orthopedic applications

Bone repair represents one of the most mechanically demanding scenarios in biomedical applications, where both stiffness matching and load-bearing capacity are critical for functional performance. The materials must match the mechanical properties of native bone. They must also adapt to the changing mechanical environment during bone regeneration. Conventional implants have fixed stiffness. As a result, they often cause stress shielding, poor osseointegration, or mechanical mismatch in the early stages of healing. VSMs provide an alternative approach. These materials can be injected or implanted in a soft state so that they conform to irregular defect shapes. After placement, they can stiffen in situ to provide load-bearing support. This transition from compliance to load-bearing support is especially important for minimally invasive bone repair. In such procedures, accurate placement is required, and mechanical stability must be established quickly after deployment.

**Fig. 12:**
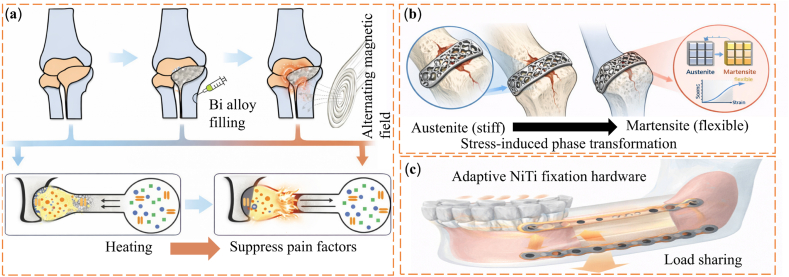
(a) Schematic illustration of Bi alloy-based bone defect filling and analgesia [279]. (b) NiTi porous bone fixation plates and tensile samples fabricated via the SLM method [280]. (c) Components of the Reconstructed Mandible [281].

LMPAs are attractive materials for minimally invasive bone defect repair because they can be injected, undergo a clear solid-liquid phase transition, and provide metal-level load-bearing strength. He et al. [279]. reported a Bi_35_In_48_._6_Sn_16_Zn_0_._4_ alloy that can be injected into bone defects in the liquid state and then solidify rapidly in vivo, as shown in Fig. 12a. After solidification, the alloy forms a scaffold that closely matches the shape of the defect. Micro-computed tomography (micro-CT) performed 210 days after implantation showed good local retention and effective bone integration. No obvious migration was observed, indicating that the alloy maintains structural stability over long periods in vivo. This approach reduces the need for bulky implants or additional fixation devices and highlights the potential of LMPA-based systems as moldable, self-sealing, and load-bearing bone substitutes for minimally invasive bone repair.

SMAs, particularly NiTi alloys and SMPs, provide another route to stiffness-adaptive bone fixation. These materials can be implanted in a compact shape and then transform into load-bearing structures after activation. This enables fixation devices to adapt to both surgical placement and post-implantation mechanical demands. Jahadakbar et al. [280]. fabricated stiffness-tunable NiTi bone fixation plates using three-dimensional printing combined with controlled heat treatment, as shown in Fig. 12b. By adjusting the plate geometry and porosity, the resulting stiffness was matched to that of cortical bone. This matching reduced stress shielding and supported more physiological load transfer. In later work, Jahadakbar et al. [281]. developed a systematic manufacturing framework for shape memory and superelastic NiTi components. In this framework, designed porous architectures enabled precise tuning of mechanical behavior to satisfy patient-specific biomechanical requirements, as illustrated in Fig. 12c. Together, these studies show that combining structural design with phase-transition-based mechanisms can produce bone fixation systems that are both mechanically adaptive and compatible with native bone mechanics.

In bone repair applications, LMPA bone cements and porous NiTi plates address different but complementary clinical needs. LMPA-based systems perform well in injectability and shape conformity. They can fill irregular and enclosed bone defects and provide clear radiographic contrast, which makes them suitable for minimally invasive repair procedures. However, long-term use may raise concerns related to metal ion release, local thermal effects during activation, and compatibility with repeated imaging procedures, especially magnetic resonance imaging (MRI). Porous NiTi fixation plates, by contrast, offer stiffness levels close to those of native bone. They also provide superelastic load sharing and good compatibility with medical imaging. These features make them suitable for long-term fixation in load-bearing bones. At the same time, their implantation usually requires more invasive surgical procedures, and they cannot be used for injection-based filling of complex or irregular bone cavities. As a result, porous NiTi fixation plates are generally preferred for load-sharing fixation in weight-bearing skeletal regions, while injectable LMPA materials are more appropriate for minimally invasive repair of enclosed bone defects. From a design standpoint, choosing between these two approaches depends on how each application balances the need for minimally invasive delivery against long-term mechanical stability and imaging compatibility at a given anatomical site.

Although variable stiffness strategies address key challenges in bone reconstruction, their translational bottlenecks differ substantially at the mechanism level. For LMPA-based systems, the primary limitation is not mechanical strength but long-term biological safety, including potential ion release, encapsulation stability, and compatibility with sterilization and repeated imaging. Their clinical pathway therefore depends on rigorous toxicological validation and reliable containment strategies. In contrast, porous NiTi fixation plates face fewer material-safety concerns but encounter manufacturing and regulatory complexity associated with additive fabrication, transformation temperature control, and long-term fatigue behavior under cyclic physiological loading. From a translational perspective, stiffness-adaptive metallic fixation systems that build upon existing orthopedic implant standards may currently have a clearer pathway toward clinical adoption, as they extend familiar surgical workflows rather than introducing entirely new injectable paradigms. Injectable phase-change systems remain highly promising for minimally invasive repair, yet their long-term regulatory acceptance will depend on demonstrating stable encapsulation and predictable in vivo performance over extended time scales. While bone represents a load-bearing extreme, the broader challenge lies in aligning stiffness-regulation mechanisms with application-specific biomechanical constraints.

Taken together, the central bottleneck in variable stiffness design lies not in achieving tunable stiffness itself, but in ensuring long-term mechanical reliability, biological stability, and regulatory feasibility under application-specific constraints.

### 4.6 Cross-domain insights and translational considerations

By synthesizing the biomedical applications discussed in this section, several cross-domain patterns emerge that differ from the mechanism-focused comparison in Section 3. In deployable medical systems, the selection of stiffness regulation strategies is governed less by intrinsic material properties than by application-specific constraints. Accordingly, VSM design is better understood as the outcome of an interplay between regulation mechanisms and clinical requirements. Once factors such as the anatomical site, activation mode, and safety limits are defined, the feasible design space is often narrowed to only a few viable strategies. At a general level, different biomedical domains tend to favor different stiffness regulation strategies. Drug delivery systems typically prioritize safety, compatibility with the gastrointestinal environment, and remote activation, which makes noninvasively triggered phase-change systems particularly attractive. Wearable devices place greater emphasis on conformability, low weight, and long-term comfort, making jamming-based architected textiles especially suitable. MIS instruments, by contrast, must navigate tortuous anatomical pathways while delivering precise forces at the target site, and therefore often rely on hybrid solutions that combine phase-change elements with antagonistic actuation. Sensing platforms and bone repair systems benefit more directly from the structural stability, tunable stiffness, and solid-liquid phase transition behavior offered by LMPAs and SMPs. These tendencies become more evident in specific use cases. These observations also reflect a general design principle of hybrid variable-stiffness systems. Different stiffness modulation mechanisms are combined to exploit complementary functionalities, such as combining compliant navigation with load-bearing support, or integrating rapid actuation with stable structural reinforcement. As a result, hybrid systems can achieve performance that is difficult to realize with a single mechanism alone, demonstrating clear synergistic effects in terms of mechanical adaptability, functional integration, and application-specific optimization. Gastrointestinal drug delivery, for example, requires remote activation under strict thermal safety limits and therefore naturally favors capsule systems based on LMPAs or SMPs activated by magnetic fields or ultrasound. Jamming structures and magneto- or electrorheological fluid systems are generally less suitable in this context because of constraints related to device size and field safety. In contrast, rehabilitation braces intended for long-term daily use must remain breathable, lightweight, and low profile. In such cases, practical designs often combine chain-mail-like or laminar jamming textiles with mechanical antagonistic structures, whereas fully encapsulated MR or ER fluid systems are typically avoided because their mass, limited breathability, and long-term safety concerns are difficult to reconcile with continuous wear.

**Fig. 13:**
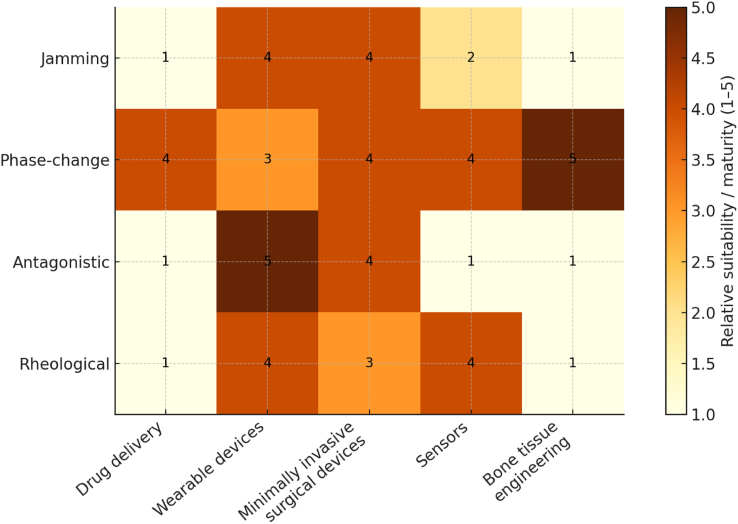
Relative suitability of four stiffness-modulation mechanisms (1-5) across five biomedical domains, based on published device-level evidence.

These application-level trends are further consolidated in Fig. 13, which maps the major stiffness regulation mechanisms onto representative biomedical domains. Rather than ranking mechanisms solely by intrinsic material properties, the heat map reflects their relative suitability and technological maturity within specific application contexts based on published device-level studies. The relative scores (1-5) capture the extent of reported implementation and technical validation in the surveyed literature. The comparison shows clear patterns. Phase-change systems are most used in drug delivery, sensing platforms, and bone tissue regeneration. Antagonistic actuation shows clear advantages in wearable devices and continuum robotic systems. Importantly, these patterns suggest that mechanisms with structurally simple architectures and clinically established materials currently present a lower translational barrier than systems requiring repeated thermal cycling, sealed fluid chambers, or embedded field hardware. In practice, their adoption is often constrained by packaging complexity and long-term stability concerns rather than by insufficient stiffness performance. Together, these trends reinforce the application-driven nature of mechanism selection in biomedical VSM design.

While these application-driven patterns provide a useful basis for mechanism selection, successful clinical translation depends on a broader set of factors that extend beyond stiffness tunability alone. In MIS, for example, catheters based on LMPAs and tendon-SMA hybrid systems can switch effectively between compliant and rigid states in laboratory settings and perform well in standard benchtop tests. However, these systems have not yet become part of routine clinical practice. The main barriers are related to sterilization, failure behavior, and integration with existing surgical workflows. In practice, sterilization compatibility is a critical but highly mechanism-dependent constraint. It can strongly influence performance retention after commonly used procedures such as autoclaving (high-temperature steam) and ethylene oxide (EtO) treatment. For LMPA-based systems, the stability of alloy distribution and, more critically, encapsulation integrity must be maintained after sterilization-related thermal or chemical exposure, as minor interfacial degradation may compromise stiffness repeatability, as reported in variable-stiffness catheter and robotic systems using low-melting-point alloys [63]. In SMP systems, sterilization procedures may shift the programmed transition temperature or affect cyclic recovery stability, thereby altering the effective stiffness window over repeated use and potentially reducing stiffness-switching performance after repeated processing cycles [282]. Jamming-based architectures are typically less sensitive at the bulk material level, yet their functionality depends strongly on membrane durability and sealing reliability; degradation of these interfaces can reduce attainable stiffness contrast in subsequent cycles, particularly after repeated sterilization or cleaning processes [63]. Antagonistic mechanisms built on cables, springs, or elastic backbones are structurally robust, but repeated sterilization exposure may induce creep or fatigue in compliant elements, gradually affecting force transmission consistency. For MR/ER systems, long-term stability of functional fluids and sealed chambers becomes critical, as any change in fluid homogeneity or containment directly influences field-responsive behavior. Clinical users and regulatory agencies therefore require clear evidence that repeated processing does not degrade encapsulation layers or compromise mechanical integrity, that the device remains safe if intraoperative heating is interrupted, and that additional components such as cables, control units, or external modules do not disrupt established instrument layouts or team coordination in the operating room. Collectively, these observations show that sterilization-induced performance decay is not uniform across VSMs but instead follows mechanism-specific failure pathways: phase-change systems are primarily limited by encapsulation stability, polymer-based systems by thermomechanical shifts, and jamming architectures by membrane durability.

Beyond sterilization and reliability, each biomedical domain also introduces its own material-specific trade-offs that shape the practical feasibility of variable-stiffness systems. In bone repair systems, injectable LMPA bone cements and porous NiTi fixation plates present a different set of trade-offs. Injectable LMPA systems offer good conformity to defect geometry, high radiopacity, and minimally invasive delivery. At the same time, concerns remain regarding long-term metal ion release and compatibility with MRI. Porous NiTi fixation plates provide stable, bone-matched mechanical support, but usually require more invasive implantation and allow limited adjustment after placement. Wearable systems impose additional constraints. Devices intended for long-term use must remain breathable and have a low profile over thousands of loading cycles. Membrane-encapsulated jamming structures can provide good initial comfort, but they may be more difficult to maintain over long periods than purely textile-based antagonistic structures, particularly in terms of durability and hygiene. Sensing platforms face yet another set of challenges. They must maintain a stable baseline under changes in temperature, humidity, and mechanical disturbance. As a result, the wide sensing ranges enabled by phase-change dielectric layers or LMPA components must be balanced against increased demands on calibration, packaging, and long-term stability. Overall, these examples suggest that the main limitations of variable-stiffness systems are often application- and workflow-dependent rather than rooted in the intrinsic capability of any single mechanism. At the same time, the clinical feasibility of different stiffness regulation strategies is also strongly influenced by the biocompatibility of the underlying material systems. Factors such as cytotoxicity, degradation behavior, and potential ion release may impose additional constraints on long-term implantation or repeated clinical use. To make these material-level considerations more explicit, Table 3 summarizes reported biocompatibility indicators for several representative variable-stiffness material systems. Overall, polymer-based systems generally exhibit favorable biocompatibility profiles, whereas metal- or particle-based systems require careful consideration of ion release and encapsulation stability. Accordingly, stiffness regulation strategies should be evaluated not only by mechanical performance, but also by sterilization compatibility, failure tolerance, and alignment with established clinical workflows. This perspective also helps explain why MR/ER systems, despite their fast response, have seen limited adoption in implantable or long-term wearable devices, and why many MIS systems that progress toward translation continue to rely on mechanically simple, robust, and easily sterilized designs. Taken together, current clinical trends suggest that systems prioritizing reliability, integration simplicity, and regulatory compatibility are more likely to reach routine practice than architectures optimized primarily for extreme stiffness contrast.

**Table 3:** Biocompatibility indicators of representative variable-stiffness material systems.

Material system	Representative materials	Cytotoxicity	Degradation	Ion release	Ref
Low-melting alloys	EGaIn; Galinstan	Low; dependent on exposure conditions	No intrinsic biodegradation; surface oxidation may occur	Possible Ga/In ion release	[[283], [284], [285], [286], [287]]
Shape memory polymers	Polyurethane-based SMPs, PCL-based thermoplastic SMPs	Generally low cytotoxicity and good biocompatibility	Biodegradable depending on polymer composition	Not applicable	[[288], [289], [290], [291]]
Magnetorheological fluids	Iron particle-silicone oil suspensions	Generally low cytotoxicity when well encapsulated	No intrinsic biodegradation; stability depends on carrier fluid	Possible iron particle leakage	[[292], [293], [294]]
Electrorheological fluids	Dielectric particle-silicone oil suspensions	Limited biocompatibility data	No intrinsic biodegradation; stability depends on carrier fluid	Possible particle leakage	[295,296]

The implications of these application constraints at the device level are further illustrated in Table 4, which summarizes representative systems across the biomedical applications reviewed in this work. For each system, the table links the application scenario to the corresponding stiffness regulation mechanism, core function, and key engineering challenges. In addition, factors such as manufacturability, long-term durability, sterilization compatibility, and regulatory requirements should be considered from the earliest stages of system design. These analyses support the central conclusion of this review: future biomedical VSMs should be developed through an application-driven, mechanism-supported co-design strategy. In this framework, the constraints of the target clinical scenario define the feasible design space and guide the selection among jamming-based, phase-change, rheological, and antagonistic mechanisms. This approach moves beyond optimization based only on material properties and enables variable stiffness systems to advance from proof-of-concept studies toward deployable, controllable, and clinically relevant technologies.

**Table 4:** Summary of representative biomedical applications enabled by variable-stiffness mechanisms.

Application Domain	Representative System/Device	Mechanism	Core Functionality	Practical Challenges	Ref
Drug Delivery	Magnetoactive capsule for triggered drug release	Smart material (Phase-change)	On-demand actuation enabling programmable, retrievable dosing	Precise release control; leakage prevention; alloy biocompatibility and thermal-safety management	263
Drug Delivery	HIFU-triggered release	Smart material (Phase-change)	Localized, reversible on-off drug release tied to thermal shape recovery	Control of dose kinetics; cavitation effects; long-term biocompatibility	264
Drug Delivery	Magnetic bio-inspired soft carrier	Smart material (Phase-change)	Body-temperature morphing, magnetic navigation, and localized dosing	Need for biocompatible coatings; targeting/retrieval variability; dose stability; limited stiffness	265
Wearable device	Hybrid exoskeleton glove	Jamming and antagonism	Variable-stiffness assistance with finger abduction and enhanced grasp force	Bulky pumps/power pack; actuator thickness; precision force control; grasping small objects remains limited	268
Wearable device	Smart bandage	Smart material (Phase-change)	Rapid self-heating expansion for on-site fixation and adaptive stiffness	Reaction control; hydrogen-related safety; reduced reusability; limited biocompatibility precision	269
Wearable device	Shape-adaptive wearable attachment	Jamming	Conformable attachment that locks via vacuum jamming to increase lateral stability	Airtight sealing; performance sensitive to contact/fit; comfort-stiffness trade-off	270
Minimally Invasive Surgical Devices	Submillimeter continuous variable-stiffness catheter	Smart material (Phase-change)	Segment-wise stiffness switching between compliant and rigid states, enabling flexible navigation and stable positioning; high load capacity and fast response	Strict thermal constraints; encapsulation for safety; slow cooling recovery; limited clinical validation	59
Minimally Invasive Surgical Devices	Bioinspired handheld time-shared continuum robot	Antagonism and smart material	Single-motor actuation of multi-DOF modules for lightweight endoscopic manipulation	Limited actuation speed (time-sharing); SMA heat load; sterilization and integration challenges	272
Minimally Invasive Surgical Devices	Soft manipulators	Fiber jamming	Large reversible stiffness tuning with shape-locking and passive safety	Airtight vacuum system; hysteresis/creep; limited bandwidth; membrane durability; added tubing mass/noise	273
Sensor	Adaptive robotic skin based on gallium microgranules	Smart material (Phase-change)	Adaptive stiffness modulation enabling ultra-wide pressure range and self-healing	Complex microgranule processing; limited long-term structural stability	69
Sensor	SMA-actuated non-bonded piezoelectric bone sensor	Smart material (Phase-change)	Impedance-based healing monitoring with adjustable, stable contact force	SMA heating and biocompatibility control; drift calibration; motion artifacts; miniaturization & sterilization	278
Bone Repair and Orthopedic Applications	Bone defect repair & magnetothermal analgesia	Smart material (Phase-change)	Long-term defect filling with strong osseointegration and radiopacity; AMF-driven local analgesia	Precise thermal control; long-term ion-release and load-bearing stability; AMF hardware requirements	198
Bone Repair and Orthopedic Applications	Mandibular/craniofacial fixation & reconstruction	Smart material (Phase-change)	Bone-like stiffness and superelastic compression for stable fixation	SLM process precision; non-uniform transformation temperatures; powder-removal difficulty; stability of superelasticity	199
Bone Repair and Orthopedic Applications	Mandibular reconstruction & fixation	Smart material (Phase-change)	Porosity-tuned stiffness (∼12 GPa) and superelastic plateau for reduced stress shielding	Control of NiTi transformation behavior; SLM defects/porosity uniformity; long-term biocompatibility	200

**Fig. 14:**
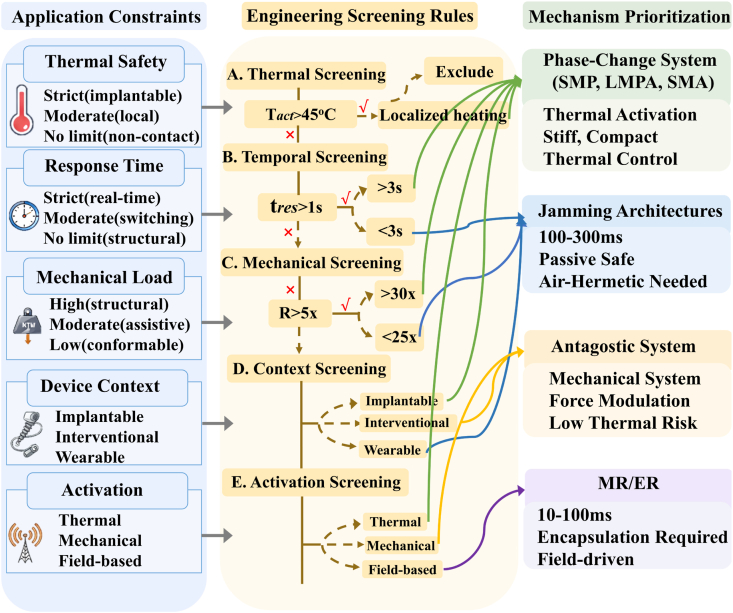
Constraint-based decision framework for mechanism selection in biomedical variable stiffness systems.

To provide a structured approach for navigating these multifaceted and often interdependent constraints, we propose the following mechanism selection framework (Fig. 14). The framework converts application-level constraints into sequential engineering screening steps, including thermal screening, temporal response evaluation, mechanical performance assessment, device context analysis, and activation feasibility. Each stage incorporates representative quantitative thresholds derived from the literature survey, reflecting typical device-level performance ranges reported in prior biomedical implementations. For instance, thermally sensitive scenarios impose strict temperature limits, typically below approximately 45 °C in tissue-contact applications. In contrast, real-time interaction tasks tend to favor antagonistic mechanisms or magneto- and electrorheological systems capable of sub-second response. Within this framework, the parameter R denotes the stiffness ratio between the rigid and compliant states. Through this structured decision pathway, phase-change systems, jamming architectures, antagonistic mechanisms, and MR/ER systems are not treated as intrinsically superior or inferior, but rather as options that become preferable under specific combinations of constraints. These mappings reflect prevailing engineering practices observed across biomedical implementations rather than fixed one-to-one correspondences, while also acknowledging that performance may vary with material formulation, structural configuration, and system-level optimization. Collectively, these constraints define the key design boundaries that must be addressed to translate variable-stiffness materials from laboratory prototypes into clinically deployable biomedical systems.

## 5 Outlook

**Fig. 15:**
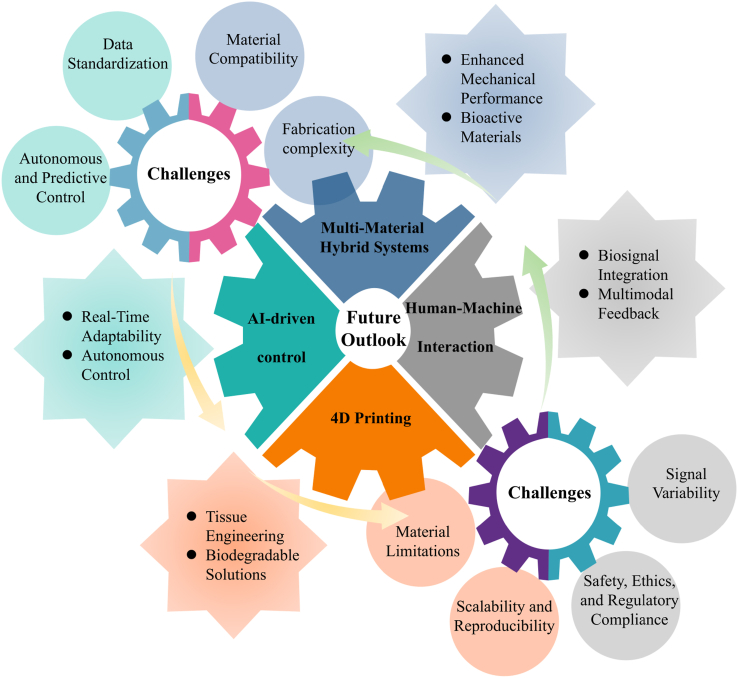
Opportunities and challenges for advancing VSMs toward biomedical applications. Polygons highlight potential advantages while circles indicate key challenges.

The rapid progress of VSMs has significantly expanded their biomedical applications, yet long-term translation will require advances that go beyond stiffness modulation alone. Despite the diversity of mechanisms reviewed above, several technical challenges remain, including limited response speed in some material systems, integration complexity in multifunctional devices, reliability under repeated activation cycles, and constraints related to clinical translation. Future work should therefore aim to transform the current catalogue of mechanisms into quantitative design rules that explicitly link stiffness-modulation strategies to application-specific constraints. Addressing these challenges will likely require coordinated progress in several interconnected research directions. As summarized in Fig. 15, future research will likely focus on four tightly interconnected directions that aim to address both engineering limitations and clinical translation challenges: intelligent control, human-machine integration (HMI), 4D-printed architectures, and multi-material hybrid systems.

1) Intelligent control: To address the control complexity and real-time regulation challenges of variable stiffness systems, AI-driven control strategies will become increasingly important as biomedical devices demand real-time, context-aware regulation of stiffness. Machine learning models, adaptive observers, and predictive control methods can estimate user intent, anticipate biomechanical loads, and modulate stiffness distributions with millisecond-level response times. These capabilities enable personalized, task-specific mechanical responses rather than fixed or preprogrammed stiffness profiles, thereby strengthening the link between clinical tasks and stiffness regulation strategies. To move beyond purely data-driven approaches, physics-informed neural networks (PINNs) offer a promising framework for modeling the coupled nonlinear, hysteretic, and time-dependent behaviors inherent in many variable stiffness mechanisms [297]. By embedding governing physical constraints, such as constitutive relations, thermo-mechanical coupling, or phase-transition dynamics, directly into neural network training, PINNs can improve prediction robustness under limited data conditions. This is particularly relevant for mechanisms exhibiting hysteresis, rate dependence, thermal diffusion delays, or pressure-stiffness nonlinearities, where the effective mechanical response depends not only on instantaneous inputs but also on loading history and internal state evolution. Hybrid physics data models offer a structured pathway toward real-time state estimation and hysteresis compensation, although their clinical viability will depend on computational efficiency and regulatory interpretability [298]. Such intelligent frameworks can translate task requirements and safety constraints into feasible operating conditions for jamming-based, phase-change-based, or antagonistic mechanisms. For instance, gastrointestinal drug delivery requires remote activation within a narrow thermal safety window; data-informed models can map this requirement to appropriate mechanisms and parameter settings, enabling context-aware stiffness control while maintaining thermal compliance. At the same time, reliable deployment depends on high-quality physiological data, consistent acquisition protocols, and computationally efficient architectures compatible with the power limits of wearable and implantable devices. Future research should therefore emphasize lightweight models, robust multisensor fusion, and interpretable decision processes to ensure safety, reliability, and regulatory acceptance.
2) HMI: To improve usability and user-device coordination in assistive and surgical contexts, future biomedical devices will require smooth two-way interaction between users and variable stiffness systems. These systems need to combine biosignal interfaces, such as electromyography (EMG), electroencephalography (EEG), and mechanomyography (MMG), with mechanical and haptic sensing inputs. At the same time, they should provide feedback through channels such as haptic cues, proprioceptive signals, and augmented reality (AR) displays. Together, these interaction pathways can improve intuitive control of assistive devices and increase dexterity and precision in robot-assisted surgical tasks. From a design viewpoint, reliable human-machine interfaces (HMIs) are essential for enabling VSMs to switch between compliant and supportive modes in line with user intent. In long-term rehabilitation braces, for example, comfort and breathability are key requirements. These constraints often make chain-mail-like or laminar jamming textiles more suitable than sealed magnetorheological or electrorheological fluid systems. In such cases, effective stiffness modulation depends strongly on accurate intent sensing and stable closed-loop feedback. At the same time, biosignals vary widely between users and are sensitive to drift and motion-related noise. These factors remain major obstacles to reliable long-term operation. Progress in human–machine interaction for variable stiffness systems will therefore depend on improved feature extraction methods, user-specific adaptive calibration, and well-defined safety and regulatory frameworks. These elements are necessary to ensure stable, controllable performance in real clinical settings.
3) 4D-printed architecture: To overcome current limitations in material responsiveness and device integration, 4D printing and stimulus-responsive structures offer a parallel route toward programmable variable stiffness systems. Unlike conventional 3D printing, where the printed structure remains mechanically and geometrically fixed after fabrication, 4D printing introduces a time dimension in which printed structures undergo programmed changes in shape or mechanical properties when exposed to external stimuli such as temperature, moisture, magnetic fields, or light. By integrating LMPAs, SMPs, hydrogels, or magnetically responsive components into additively manufactured structures, implants, scaffolds, and microrobots can change both shape and stiffness in vivo according to predefined rules. For example, recent studies have demonstrated thermally triggered shape-morphing structures fabricated from shape-memory polymers that can be minimally implanted and subsequently expand to a programmed configuration at body temperature [299]. More recently, magnetically or thermally responsive 4D-printed biomedical structures have been reported to enable programmable shape transformation and stiffness modulation after implantation, highlighting the ability of printed architectures to evolve over time under external stimuli. In another example, hydrogel-based 4D-printed scaffolds have been shown to undergo swelling-induced deformation in aqueous environments, enabling time-dependent structural transformation and tunable mechanical behavior for tissue engineering applications [300]. This approach is especially relevant for tissue engineering, MIS, and adaptive load-bearing applications. When combined with application-aware design strategies, 4D-printed systems can incorporate embedded response rules. For example, an implant may stiffen automatically as local mechanical loads increase or as tissue healing progresses. In this way, mechanical performance can adapt over time to match the surrounding physiological environment. However, current 4D variable stiffness systems still face major challenges. These include limited biocompatibility of some smart materials, slow or inconsistent response speed, reduced performance after repeated activation cycles, and strong sensitivity to in vivo environmental variations. As a result, most reported systems remain at proof-of-concept stage and have not yet demonstrated long-term in vivo reliability. Together, these factors limit clinical translation. Future research should therefore focus on developing biointegratable smart materials, improving the accuracy and repeatability of multimaterial additive manufacturing, and establishing predictive computational design tools. These efforts are required to enable reliable, repeatable, and clinically relevant 4D variable stiffness structures.
4) Multi-material hybrid systems: To resolve structural and biocompatibility trade-offs in biomedical environments, multimaterial and hybrid structures offer a complementary approach for achieving spatially graded stiffness, improving mechanical robustness, and supporting biological integration. By combining metals, polymers, hydrogels, and ceramics within a single architecture, these systems can better reflect the mechanical heterogeneity of native tissues. In such designs, different materials are intentionally assigned specific functional roles. Metals typically provide load-bearing capacity and structural stability, polymers and hydrogels enable compliance or adaptive stiffness regulation, and bioactive ceramics promote biological integration with surrounding tissues. This matching helps reduce stress shielding and may extend the service life of implants. However, long-term durability is still limited by problems such as weak interfacial bonding, layer separation, and mismatch at the microstructural level. Progress toward long-term biomedical use will therefore depend on advances in interfacial chemistry, surface modification, and precise control of material placement at small length scales. From a mechanism-application co-design viewpoint, added structural complexity is justified only when it resolves a clear engineering trade-off. In bone tissue engineering, for example, porous NiTi fixation plates and injectable LMPA bone cements address different needs. NiTi plates support load sharing and mechanical stability, while LMPA cements offer good radiopacity and conformal filling of defects but raise concerns related to ion release. Their complementary roles illustrate how multimaterial hybrid strategies can be effective when they are chosen to meet specific application requirements. Together, these materials provide complementary mechanical and functional advantages, demonstrating how hybrid strategies can achieve performance that cannot be obtained with a single material system.

At present, the most realistic path toward clinical deployment lies not in maximizing stiffness contrast, but in improving reliability, manufacturability, and integration compatibility within existing surgical ecosystems. Finally, several cross-cutting challenges continue to limit the clinical translation of variable stiffness systems. Addressing these challenges will require closer collaboration between materials scientists, biomedical engineers, clinicians, and regulatory experts. These challenges include sterilization compatibility, scalable manufacturing, long-term stability, and regulatory approval. Addressing them requires early and coordinated co-design across materials science, biomechanics, control engineering, and clinical practice. From a translational perspective, most current biomedical VSM implementations remain at the laboratory or prototype validation stage, corresponding to early technical readiness. Progress toward clinical deployment will likely follow a staged translational pathway broadly consistent with increasing technical readiness levels: (i) benchtop verification of mechanical reliability and repeatability, (ii) validation in relevant biological environments with emphasis on sterilization compatibility and biocompatibility stability, (iii) preclinical large-animal studies assessing safety, durability, and failure behavior, and (iv) integration into regulated clinical workflows with manufacturing scalability and documentation compliance. In this staged process, stiffness regulation mechanisms should be developed together with constraints related to sensing, navigation, sterilization, and clinical workflows, rather than being optimized as isolated subsystems.

In the longer term, the integration of smart materials, multimodal actuation, and intelligent control is expected to shift VSMs from single-function components toward system-level clinical platforms. The core design principle is straightforward. Under clearly defined anatomical conditions, surgical procedures, and regulatory constraints, designers should select only the mechanisms that are necessary to achieve safe, reliable, and clinically useful performance. This means choosing an appropriate combination of jamming-based, phase-change-based, rheological, and antagonistic strategies, instead of increasing mechanism complexity without clear clinical benefit.

## CRediT authorship contribution statement

**Jiawen Xu:** Conceptualization, Investigation, Methodology, Writing – original draft, Writing – review & editing. **Run Lin:** Conceptualization. **Dongfei Huo:** Conceptualization. **Junyan Li:** Methodology. **Oana R. Ghita:** Writing – original draft, Writing – review & editing. **Ken E. Evans:** Writing – original draft, Writing – review & editing. **Xijin Hua:** Conceptualization, Funding acquisition, Methodology, Project administration, Supervision, Writing – original draft, Writing – review & editing.

## Declaration of competing interest

The authors declare that they have no known competing financial interests or personal relationships that could have appeared to influence the work reported in this paper.

## Data Availability

Data will be made available on request.
